# Impact of ion intercalation materials on advancing capacitive deionization: from theory to practical

**DOI:** 10.1039/d5na00311c

**Published:** 2025-06-19

**Authors:** Mahima S., Manjunatha Kumar K. S., Prajwal Sherugar, Xuezhu Xu, Gurunatha K. L., Mahesh Padaki, D. H. Nagaraju

**Affiliations:** a Department of Chemistry, School of Applied Sciences, REVA University Yelahanka Bengaluru Karnataka 560064 India nagaraju.dh@reva.edu.in +91 9900634435; b South China Academy of Advanced Optoelectronics, South China Normal University China; c Centre for Nano and Material Sciences, JAIN (Deemed-to-be University) Jain Global Campus, Kanakapura, Ramanagaram Bangalore 562112 India sp.mahesh@jainuniversity.ac.in +91 9538414994

## Abstract

Capacitive deionization (CDI) is an innovative technology that relies on the electrostatic adsorption of ions onto the electrode surface. Recently, the use of ion intercalation materials has been shown to be a viable method for increasing electrosorption capacity, which can greatly improve CDI performance. In this review, the most recent developments in ion intercalation, synthesis techniques, electrode performance, applications, and cell design in CDI systems are examined. Furthermore, this review highlights the economic feasibility, cost-effectiveness and development of technologies that use seawater sources to produce drinkable water compared with traditional desalination methods. Additionally, it draws attention to the function of advanced electrode materials in CDI, which highlights the possibilities of composite engineering for increased desalination efficiency. It also discusses the development of reliable and scalable CDI systems with improved capacities for environmentally friendly water filtration.

## Abstract 2

Figure highlights the synthesizing strategies, properties, applications, advantages and strength of ion intercalation materials by understanding their different behaviour.

## Graphical abstract

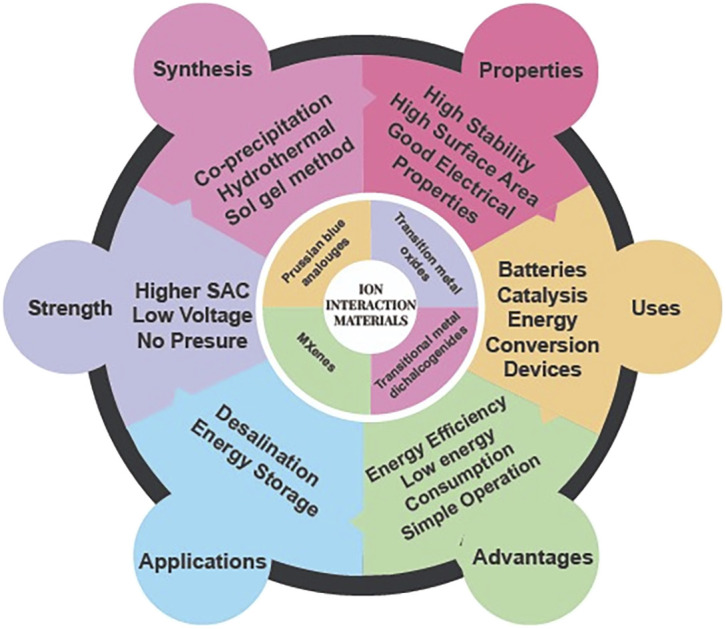

## 1. Introduction

Water scarcity is a major concern in India due to population growth, increased urbanization, industrial expansion and the effects of climate change. Therefore, access to clean drinking water has grown challenging because of groundwater depletion, declining freshwater supplies, and a rise in contamination from natural sources. The United Nations' World Water Assessment Program (WWAP) report (2017) predicted that by 2030, the world will face a water shortfall of 40% unless resource management improves significantly.^1^ To address this issue, there are several water purification techniques, among which reverse osmosis (RO) is one of the most widely used for the water filtration process. The system operates on the principle of applying high pressure to pass water through a semi-permeable membrane, which selectively allows water molecules to pass while blocking dissolved salts, contaminants and impurities. Utilizing seawater resources for producing drinking water *via* the RO method is imperative; therefore, several RO plants have been installed to produce desalinated water. Nevertheless, RO treatment has some disadvantages despite its effectiveness, such as excessive water waste, high energy consumption, and fouling.^2–4^ Because of these drawbacks, researchers have been looking into different purifying methods, including capacitive deionization (CDI), which is an electrochemical technique that selectively eliminates salt ions by forming an electrostatic double layer (EDL) on the surface of electrodes.^5^ Among the array of emerging methodologies, CDI offers a cost-effective solution for brackish water desalination, in which ions with a positive charge are drawn towards a negative charge electrode and *vice versa*, aiding in the removal of ions from feed water without the use of high-pressure pumps.^6^

**Fig. 1:**
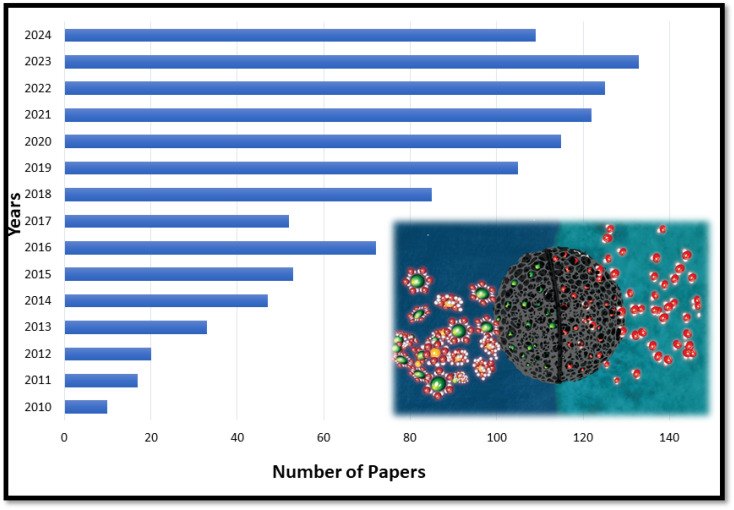
Graphical representation of the number of papers published on CDI over the past 12 years to date.

CDI has emerged as a promising alternative because it offers an expanded range of ion selectivity for cation removal compared with electrodialysis (ED).^7^ Porous electrodes constitute a critical component in the CDI process, prompting ongoing research efforts aimed at developing efficient electrode materials that present numerous advantages over membrane technology, antifouling, elimination of chemical usage in the process, simplified maintenance, time efficiency, reusability, extended operational lifespan, and reduced process costs.^8,9^ Therefore, CDI operates on the principle of adsorbing charged ions onto the electrode from the inlet feed, allowing fresh water to move towards the outlet feed, while absorbed ions subsequently desorb in the next stage, producing concentrated effluent called brine.^10^ CDI research has gained momentum since the publication of pioneering articles in 2013 and 2014,^11,12^ followed by further advancement in 2015.^8^ Additionally, Welgemoed *et al.*^9^ explored the development and evaluation of capacitive deionization technology (CDT) as a promising alternative to conventional desalination methods such as RO and ED. Their study highlighted the superior energy efficiency, consuming only 0.594 kW h m^−3^—significantly less than traditional methods—and underscored its potential for scalability and automation in industrial applications. Their findings suggested that CDT could be a key solution in addressing global water scarcity by providing an effective approach to desalinating brackish and seawater sources. The development of electrode materials capable of adsorbing ions at low potential remains a major challenge, although recent innovations have shown improved efficiency. Therefore, CDI technology has evolved beyond desalination to encompass various water purification processes owing to advancements in electrode orientation.^13^ The number of research articles published each year has been steadily increasing (Fig. 1). This suggests that interest in this area is expanding. However, targeted research is necessary to advance useful breakthroughs in emerging challenges, such as energy consumption, long-term stability, and cost-effectiveness.

To remove ions from water, CDI uses the principle of electrostatic attraction, in which voltage is supplied across the electrodes immersed in the water solution and partitioned by a separator. The ions in the water are drawn towards the electrode with opposite charges and accumulate there due to electrostatic forces. Water is then successfully deionised by passing it over the electrodes, which removes the ions from the solution, thereby leading to purification. When the voltage is reversed, the ions that have accumulated within the porous electrode materials are discharged, facilitating system regeneration; hence, CDI involves two steps: purification and regeneration.^14^ The primary methods for storing ions in electrodes include capacitive electrosorption,^15–19^ pseudocapacitive storage^20,21^ and ion intercalation. The removed ions are retained in the diffuse region of the two electrodes, forming electrostatic double layers, also known as polarization layers. The CDI stack continuously receives a steady stream of the solution, and the ion concentration in the effluent gradually decreases compared to that in the inflowing solution. Once the polarization layers reach their maximum ion adsorption capacity, reducing the applied voltage causes the ions to be released back into the solution, generating a highly concentrated ion product stream. Ions are not only removed from the solution *via* surface adsorption but also by the intercalation of ions, which leads to a high salt adsorption capacity (SAC mg g^−1^).^22^ When the ions are intercalated into layered materials,^23^ the space between the unit cells is increased and the coupling is decreased. Thus, intercalation can change the crystal and electrical structures of the host materials and is beneficial for capacitive deionization by increasing the surface area of the materials. Ion accessibility, stability and decreased coupling between layers, the diffusion barriers are lowered, hence increasing the desalination rates and better overall performance is made possible by improving the ion transport.^24^ In the literature, the specific energy consumption of brackish water reverse osmosis was reported to be around 0.5–3 kW h m^−3^,^25,26^ and it does not require high-pressure pumps, expensive membranes or secondary regeneration wastes. The main ways in which CDI varies from other water treatment methods are in its ion removal mechanism and efficiency, which uses electrical energy to function and makes it more efficient than conventional techniques like distillation or RO. Moreover, it can be developed with flexibility in decentralized water treatment applications due to its scalability and modular design.

Highly porous materials^27,28^ are frequently utilised for the electrosorption of ions in CDI and Membrane CDI (MCDI) because of their exceptional chemical, mechanical and thermal stabilities and high specific surface area. Carbon-based materials such as graphene,^29^ carbon nanotubes,^30,31^ ordered mesoporous carbon,^32^ carbon aerogels^18,33^ and hierarchically porous carbon^34^ are examples of highly porous materials as they have the highest accessible surface area and the best electrical conductivity, making them the most explored electrode materials in the CDI sector.^35,36^ In CDI devices, the electric charge at the electrodes is proportional to the energy expended during ion removal, and the charge efficiency can be crucially evaluated by modifying the carbon surface to prevent co-ion ejection,^37^,^38^ which can be altered by using,^27^ tetraethyl ortho-silicates,^28^ fluoride,^39^ sodium dodecyl sulphate,^40^ and permanganate ions.^41^ Furthermore, it can be achieved by materials, in the development of energy storage and in search of low-cost electrode materials, ion-intercalation materials like Prussian blue analogues (PBAs)^42–45^ and MXenes^46–48^ have attracted the attention of researchers in the field of CDI. Desalination systems based on intercalation materials also offer more flexibility for the selective removal of desired ions without undergoing any chemical alterations to the electrode. Inserting an ion, often a cation, into interstitial spots allows for ion storage in these materials. This process is favourable because it has the quick kinetics that makes carbon adsorption desirable, and while removing the co-ion repulsion, the ion storage capacity increases. Hence, this paper highlights the latest advancements in electrode materials, including their synthesis strategies, performance and different modes of CDI systems, by ensuring economic viability and cost-effectiveness compared to existing methods. This paper presents an imperative study to develop technologies that use saltwater resources as a supply of raw materials for the production of drinking water, as well as for the electrodes used in the field of desalination.

## 2. Theoretical foundation to design CDI cells for practical applications

As described in the previous section, CDI operates based on the principles derived from supercapacitors, specifically, the electrical double-layer (EDL) theory and the concept of pseudo-supercapacitors.^49^ CDI is used for both energy storage and water purification because it shares a feature with supercapacitors: the formation of an electric double layer at the electrode–electrolyte interface. This electric field attracts cations and anions from brackish water to the surfaces of oppositely charged electrodes. The process begins by charging the CDI cell with a power source. Once charged, the cell discharges by reversing the potential, separating freshwater and brackish water.

A typical CDI cell consists of several components, such as non-conductive acrylic end plates, current collectors, separators, and electrodes. The design, configuration, materials, and operating environment influence the performance and application of the CDI system. The end plates are equipped with valves for the water inlet and outlet, ensuring proper water flow and structural integrity. Current collectors, which can be made from materials like titanium sheets, graphite plates, graphite films, and stainless steel, play a crucial role in applying voltage across the electrodes. A DC power supply or potentiostat is used to provide a constant voltage to the cell. The assembly consists of alternating layers of end plates, current collectors, electrodes, and separators. For membrane CDI (MCDI), anion exchange membranes and cation exchange membranes are placed between the current collectors and spacers.^50^ The electric field generated within the CDI cell can be adjusted by varying the electrode materials and operating conditions (such as voltage and flow rates), which improves the performance of both water purification processes.

To enhance electrochemical activity, active materials can be coated or drop-cast onto substrates like carbon paper, graphite sheets, stainless steel mesh, nickel foam, or carbon cloth. These substrates provide mechanical support and influence ion transport and charge transfer kinetics, thereby affecting long-term stability, energy efficiency, and ion adsorption capacity. After cleaning, the coated substrates are placed on the current collectors. Separators are used between the current collectors to maintain even water flow while preventing electrode contact. Silicon rubber plates are often used to prevent leakage. Water is pumped into the CDI cell *via* a peristaltic pump at a controlled flow rate (typically 10 to 50 ml min^−1^) to ensure that the electrodes are evenly wetted, promoting efficient ion removal. The water conductivity was continuously monitored using a conductivity probe in the solution tank to track the ion removal process during adsorption and desorption. The voltage was maintained between 1.0 and 1.6 V and further optimized to enhance the ion removal efficiency. When an electric field is applied across the electrodes, water ions are attracted to the opposite electrodes. Once the electrodes are saturated, the polarity is reversed, allowing the adsorbed ions to desorb into the solution. All data from the process were recorded for analysis. The separators used in the CDI cell are essential parts that ensure the effective and safe functioning of the CDI cell by permitting smooth water flow and ion passage while preventing electrode short-circuiting. Through the optimization of flow dynamics and the reduction of ohmic resistance, separators enhance energy efficiency and reduce operating expenses.^51^ They play a critical role in improving the long-term dependability and durability of such systems.

The CDI system offers several advantages, including minimal secondary emissions, low energy consumption, easy operation, and cost-effectiveness. Due to these benefits, it was awarded the “People's Choice Award” by the World Association of Industrial and Technical Research Organizations in 2020. CDI is especially effective for treating brackish water, which has lower salinity than seawater, making it a promising solution for areas with freshwater scarcity. The overall efficiency of the CDI system depends on the design and construction of the CDI cell, the thickness of the current collectors, and the flow rate of the water. In 2018, Remillard *et al.*^52^ highlighted the impact of operational factors such as flow rate, hydraulic retention time (HRT), and voltage on CDI performance. They found that although higher voltage improves salt removal, it can also increase electrode oxidation and cause faradaic side reactions, which negatively impact performance. For flow-through CDI (FT-CDI) systems, they discovered that high flow rates and shorter HRT (<5 minutes) optimize performance, whereas in flow-by CDI (FB-CDI), increasing HRT and flow rate enhances salt adsorption capacity (SAC) and salt adsorption rate (ASAR). The current collector thickness plays an important role in the system's efficiency. Thicker collectors provide additional weight and resistance, whereas thinner collectors reduce the electrical resistance, improving the SAC.^53^ Koller *et al.*^54^ found that bipolar plates (BPs) made from expanded graphite demonstrated up to 55% higher salt transfer rates than graphite plates (GPs), due to better charge transport and reduced surface roughness. BPs are also more suitable for scaling up FCDI systems because of their mechanical stability, high production efficiency, and affordability. The flow rate of the peristaltic pump also affects system performance. Higher flow rates (50–100 ml min^−1^) can improve output but decrease in residence time of ions, lowering salt removal efficiency. In contrast, lower flow rates (5 to 20 ml min^−1^) enhanced the adsorption efficiency.

**Fig. 2:**
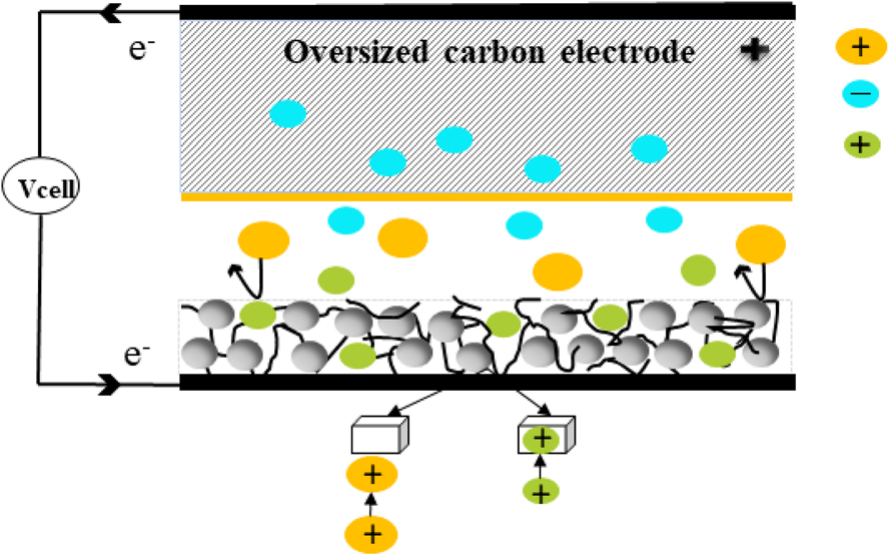
Schematic representation of the mechanism of ion interaction in an asymmetric CDI cell.

There are two main charge storage mechanisms: electrostatic interactions (electrical double-layer capacitance, EDLC) and ion intercalation. In carbon-based materials such as activated carbon, carbon nanotubes, carbon aerogels, and graphene, the EDLC mechanism is dominant. In this mechanism, applying a voltage creates an EDL at the electrode–electrolyte interface, forming a double layer. In low-dimensional materials like graphene, quantum capacitance is also important, especially near the point of zero charge (PZC), where the capacitance decreases because of the low density of states.^55^ In graphene electrodes, the electrostatic interaction between graphene and electrolyte ions shrinks the double layer, enhancing the capacitance, especially in thinner graphene electrodes. Although EDLC-based materials offer good reversibility and fast charge/discharge rates, their surface area limitations restrict their ability to adsorb significant amounts of salt. However, in ion intercalation materials, in addition to double-layer formation, the ions intercalate and de-intercalate from the crystal structure, as shown in Fig. 2, thereby boosting the salt adsorption capacity (SAC) and ion storage per unit mass. Hence, the ion intercalation process is significantly more effective than EDLC behaviour for CDI applications because of the deeper ion penetration, which leads to a more effective desalination method. For example, the cyanide-mediated mechanism of PBA and its effect on ion storage were examined in 2022 by Nordstrand *et al.*^56^ By applying a minimum voltage, the negatively charged cyanide (CN^−^) groups that aligned the holes were drawn towards positively charged sodium ions (Na^+^), stabilizing them close to the electrode walls. Similar to climbing a ladder, the ions move hopping diagonally across neighbouring CN^−^ lined faces, with each CN^−^ group serving as a step. Hence, this mechanism is called the ladder mechanism. Significant differences existed in the energy required for ion transitions, with higher barriers near defects that disrupted the framework and smaller barriers near intact CN^−^ walls. The significance of these configurations in controlling the physical characteristics of PBAs, such as storage capacity and transit efficiency, was brought to light by Simonov *et al.*^57^ using single crystals and examining their X-ray diffuse scattering patterns. Hence, the development and optimization of CDI technology are promising solutions for water purification and energy storage. Factors such as electrode material selection, current collector design, flow rate, and operational conditions play key roles in enhancing the efficiency of CDI systems. With continued research into new materials, such as ion-intercalating electrodes, CDI technology could revolutionize desalination processes, offering a more sustainable and efficient alternative to traditional methods. Therefore, the implementation of ion intercalating electrode materials has sparked interest in novel ideas for CDI because of the ion intercalating mechanism.

## 3. Experimental design, types, and modes

**Fig. 3:**
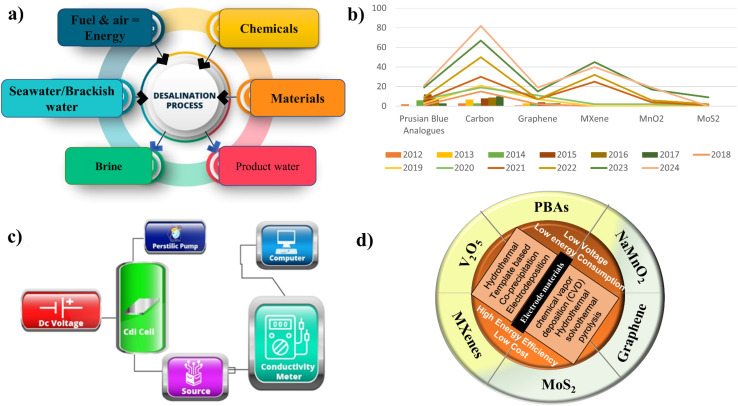
(a) Schematic of desalination process inputs and outputs. (b) Graphical representation of the number of papers published based on CDI electrode materials in the past 10 years. (c) Framework of capacitive deionization (CDI) system diagram. (d) Overview of few electrode materials for capacitive deionization (CDI): synthesis methods and key advantages.

Extensive research has been conducted to explore the elements of cell layout, electrode material design, and optimization of operational mode to enhance the ion removal efficacy of the CDI system. The ability of CDI to selectively remove particular ions is one of its main advantages; it offers adaptable water purification solutions for a range of applications. Fig. 3a illustrates the input and output of the desalination process, which uses materials (anion exchange membrane (AEM), cation exchange membrane (CEM), separator, and electrode materials), energy, and chemicals to treat saltwater to produce freshwater, with brine as a byproduct. In Fig. 3b, the graph shows the number of yearly research publications on various materials used in desalination and energy storage applications. Publications on materials like carbon, graphene, and MXenes have increased significantly over time, whereas those with ion-intercalation materials like PBA and MnO_2_ have decreased, suggesting a slower rate of research growth. However, because of their high charge storage capabilities and tuneable architectures, ion-interaction materials hold great promise for next-generation energy storage and desalination applications by strengthening their stability and refining synthesis methods.

### 3.1. Experimental design

As discussed in the previous Section 2, the desalination experiment required a CDI cell, solution tank, conductivity meter, peristaltic pump, and direct current (DC) voltage source to conduct the CDI adsorption tests, as shown in Fig. 3c. A circulation system was established to facilitate the flow of solution from the solution tank into the CDI cell and back to the tank. Throughout the experiment, the solution flow rate (mL min^−1^) was kept constant, and a DC source was employed to deliver the DC voltage. A conductivity meter attached to the computer was used to detect the conductivity of the solution in real time, and data were logged every minute. The majority of reported CDI research employs a configuration with two porous electrodes with average thicknesses ranging from 100 to 500 μm, arranged parallel to one another with a small planar gap by a separator between the electrodes through which water can flow. Fig. 3d illustrates the various materials used for CDI applications, along with their various synthesis routes and advantages for CDI cells.

### 3.2. Operational types of CDI

**Fig. 4:**
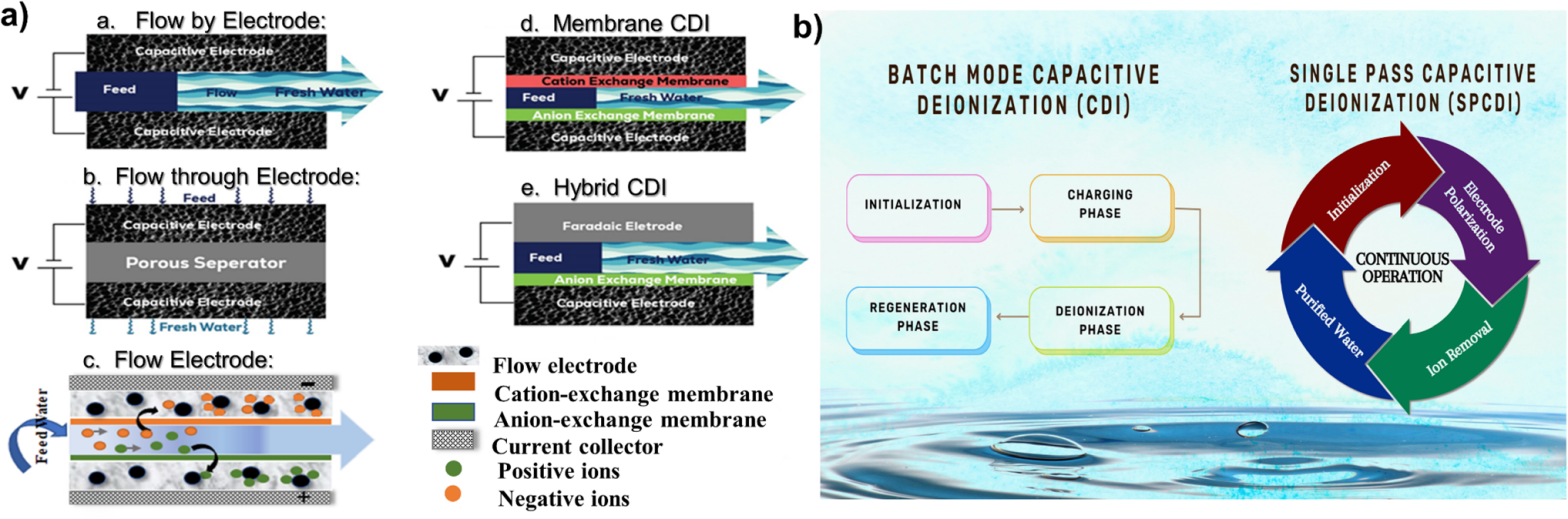
(a) Different types of CDI systems and (b) flow chart of batch and single-pass modes in CDI.

In a conventional CDI system, two porous electrodes are spatially separated by a channel that allows the flow of solution; initially, both electrodes are polarized through the application of current or voltage, while the influent solutions, containing various ions such as cations, anions, and other contaminants like minerals, salts, and organic compounds, are pumped through the channel. This polarization causes one electrode to acquire a positive charge and another a negative charge. Therefore, the oppositely charged ions in the solution are electrostatically attracted to the electrode surfaces, leading to ion adsorption and water desalination. In the second step, the applied voltage or current is reversed (or turned off), leading to the desorption of the previously adsorbed ions back into the solution. This process regenerates the electrodes and forms a concentrated effluent stream (brine), allowing continuous operation in repeated cycles.^58^ To increase overall performance, scalability, and efficiency in various applications, numerous CDI configurations have been developed throughout time. Each type offers distinct benefits in terms of energy usage, ion removal efficiency and operational adaptability. The primary types are Flow-By CDI (FB-CDI), Flow-Through (FT-CDI), Membrane CDI (MCDI), Hybrid CDI (HCDI), and Flow CDI (FCDI), as shown in Fig. 4a. The evolution of CDI has progressed with various design innovations since the 1960s to increase the energy economy and desalination effectiveness. The oldest known method is FB-CDI, which involves water flow between the stationary porous electrodes and was first demonstrated by Murphy and Caudle in 1967.^59^ Due to co-ion repulsion, this approach exhibits low charge efficiency but offers important insights into electrochemical demineralization. Subsequently, Johnson and Newman *et al.*^60^ modified the FB-CDI in 1971, which increased the effectiveness of ion removal by forcing water between the porous carbon electrodes by applying an electric field perpendicular to the direction of flow. This arrangement allowed for efficient ion adsorption through a capacitive mechanism, which made it easier for ions to travel towards their oppositely charged electrodes due to the optimized applied voltage. Then, the concept of FT-CDI was proposed, in which water flows through the porous electrodes. The first patent for this invention was filed in Canada by Andelman (CA 2444390 C) in 2002.^61^ It explained that an electrochemical desalination process involves the removal of ions by passing water over the porous electrodes. MCDI was experimentally validated by Lee *et al.* in 2006 (ref. 62) to increase the charge efficiency by introducing ion-exchange membranes into the traditional CDI. This type of CDI showed increased salt removal capacity by selectively allowing counter-ions while blocking co-ions. It became a popular type due to the better desalination performance and lower energy consumption among researchers, but it lacked charge-storage capacity. In 2014, Lee *et al.* introduced hybrid CDI to boost the charge-storage capacity of electrodes.^63^ In hybrid CDI, carbon-based materials such as activated carbon, graphene, carbon nanotubes, and carbon aerogels were used as anodes and faradaic materials such as PBAs, TMOs, and other redox-active materials as cathodes were used to utilize both charge-storage and ion-intercalation mechanisms. Therefore, a greater ion removal rate and enhanced cyclic stability were made possible with HCDI, which further minimized the gap between conventional CDI and battery-like charge storage systems. However, because of electrode deterioration, HCDI continued to exhibit limited long-term stability. To overcome the problem of electrode saturation, Jeon *et al.*^64^ in 2013 proposed FCDI as a continuous flow of suspended carbon particles rather than fixed electrodes like classic CDI; therefore, desalination can continue without the requirement of periodic electrode regeneration. The proposed model is a viable option for large-scale desalination because it can operate for extended periods and is especially useful for treating seawater and high-salinity water. Among all types of CDI, most researchers have combined the advantages of MCDI and HCDI, offering a viable strategy for high-performance CDI applications. In 2018, Santos *et al.*^65^ used carbon nanotube fiber (CNTF) combined with g-AlO_3_ and SiO_3_ as the anode and cathode, respectively. With a salt adsorption capability of 6.5 mg g^−1^ from brackish water (2.0 g NaCl/L), this design improved desalination performance. It showed a low energy consumption of 0.26 W h g^−1^ of salt removed and a high charging efficiency of 86%. In electrochemical desalination, the hybrid electrode design greatly outperformed conventional CDI systems because of its enhanced surface area optimization and decreased internal resistance. In contrast, vanadium-based intercalating materials, such as vanadium oxide (V_2_O_3_) and vanadium nitride (VN), are becoming more popular because of their strong redox activity and electrical conductivity, which improve their capacity for ion exchange and charge storage. Hence, in 2024, Wunch *et al.*^66^ showed the synergetic effect of a VN/V_2_O_3_ hybrid anode and activated carbon as the cathode, which facilitated high specific capacitance and energy density. In addition to an enhanced charge storage mechanism *via* pseudocapacitance behavior, this combination is useful for supercapacitor applications. Compared to conventional symmetric CDI electrodes, these ion intercalating materials perform much better electrochemically when employed as anodes, which in turn increases the specific capacitance and desalting efficiency.

### 3.3. Operational modes of CDI

The desalination experiments were performed in two operational modes: cyclic (Batch mode) and continuous (Single-pass mode). In the cyclic desalination process, a symmetrical cell was fed by two reservoirs containing identical solutions, and a constant positive direct current was applied until the cell voltage reached a cut-off of 1 V for 1 h and thus concludes the first step. Once the electrodes were saturated, a negative current was applied in the opposite direction until the voltage reached a cut-off of −1 V and was supplied over the cell for 1 h to saturate the electrode. This second step completes one full cycle. The only difference between the two modes of CDI is that single-pass CDI allows continuous operation, *i.e.*, feed water flows continuously through the electrodes; hence, ion removal takes place and continuously purified water is collected, making it suitable for industrial applications requiring a continual supply of cleaned water, whereas batch mode CDI is appropriate for periodic purification cycles, in which regeneration step is required and ions are removed and the regenerated electrodes are used for next cycle, as shown in Fig. 4b. However, both modes can be applied in laboratory experiments, brackish water desalination, Point-of-Use water purification, and water reclamation in Industrial settings.

The initial analysis conducted by Lado J. *et al.*^67^ revealed that the feed growth concentration of the batch mode test had a favourable effect on SAC, enabling it to achieve greater values at higher concentrations. The highest SAC and the stability of the electrode in the CDI stack were mostly discovered under batch mode; in addition, practically, all previous works in literature took an interest in SAC and concentrated on the numerous ways to enhance the SAC, which can be calculated by using eqn (1).^68^ The following formulae can be used to determine the SAC, ASRR, charge efficiency, energy consumption, and mean deionization rate.1
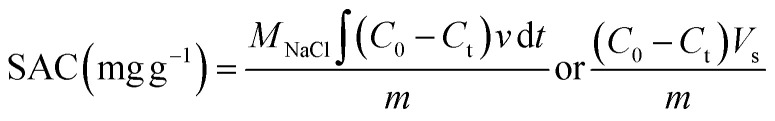
2
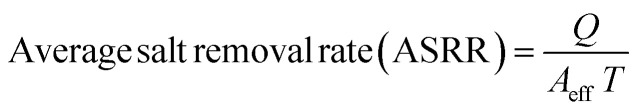
3
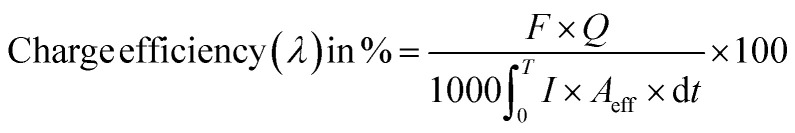
4
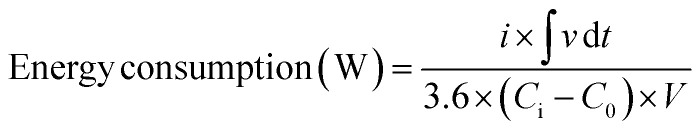
5
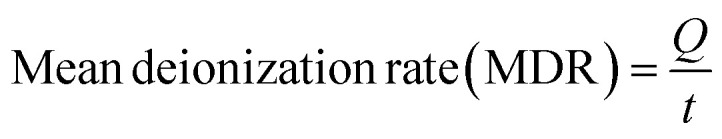
where SAC is the salt adsorption capacity (SAC in mg g^−1^), *C*_0_ (mg L^−1^) is the initial NaCl concentration of the feed water, *C*_i_ (mg L^−1^) is the NaCl concentration of the effluent water at the time *i*; *V* (L) is the volume of the treated water; *v* (mL min^−1^) is the flow rate of the feed water; *M* (g mol^−1^) is the molar mass of NaCl; *m* is the total mass of the material coated (g) and *T* (s) is the charging time, *A*_eff_ is the effective contact area between the flow electrode and the ion-exchange membrane,^68^ where *F* is the Faraday's constant, 96 485 C mol; I is current density in A m^−2^. Results showed that in batch mode, the effluent conductivity during charging declined consistently, whereas in single-pass mode, it decreased immediately and subsequently levelled out. Because of the recycling reservoir, batch mode experiments require less solution volume than single-pass experiments. In batch-mode CDI, the SAC is calculated in accordance with the variation between the initial and final salt concentrations. Because the eventual salt concentration in a recycling reservoir is a function of the total volume of the solution, batch-mode studies are limited by the unknown previous value. The single-pass mode was used with continuous feed water, and the conductivity at the leaving section of the CDI cell was measured to avoid these limitations.^69^ Recent developments include the CDI Ragone plot,^70^ which provides three crucial metrics, including the SAC, mean deionization rate (MDR), and deionization time, to evaluate the desalination performance of single-pass CDI. Because of its large surface area and low cost, AC was initially chosen as the principal electrode material for single-pass CDI systems. The electrosorption selectivity for anions was found to be NO_3_^−^ > SO_4_^2−^ > F^−^ > Cl^−^ > As, indicating that competing ions influenced arsenic removal. The single pass-CDI removed 76% of the arsenic, lowering the arsenic concentration from 0.13 mg L^−1^ to 0.03 mg L^−1^ and fulfilling the drinking water regulations. The process involved continuously pumping groundwater through the CDI cell at a flow rate of 5 ml min^−1^, where an external voltage of 1.2 V was applied during the electrosorption stage to attract and adsorb ions onto the activated carbon electrodes.^71^ After the charging phase, the voltage was removed, initiating the desorption stage, which allowed the concentrated ions to be released back into the solution. This step completed the ion removal cycle and facilitated electrode regeneration. In 2022, Zhang *et al.*^72^ investigated single-pass CDI with HNO_3_-modified activated carbon (AC) electrodes, and found a considerable increase in the fluoride removal efficiency. The improved electrodes exhibited a 13% greater adsorption capacity (3.58 mg g^−1^) and a 25% improvement in charge efficiency (22.7%) than the unmodified electrodes. The modification increased the specific surface area of AC and hydrophilicity, allowing for greater ion transport while consuming less energy. Furthermore, the modified electrodes preserved 83% of their initial adsorption capacity after five cycles, indicating excellent cycle stability. Recent advances in single-pass CDI have focused on the use of ion intercalation materials to improve desalination performance. Furthermore, Gendel *et al.*^73^ employed batch-mode CDI with carbon particle slurries as flowing electrodes (FCDI) in an electrochemical cell. During desalination, a NaCl solution is cycled through the cell, allowing ions to adsorb onto the carbon electrodes. After the desalination efficiency exceeded 99%, indicating very effective ion removal, the charge efficiency varied from 87.6% to 96%. The system efficiency in terms of ion adsorption and energy use was improved. Hence, in 2022, Jiang *et al.*^74^ used an anion exchange membrane to separate the CDI cell chamber into two, a LiMn_2_O_4_/C cathode in LiCl solution and a NaTi_2_(PO_4_)_3_/C anode in NaCl solution. When a voltage was applied, lithium ions were released from the cathode, and sodium ions were intercalated into the anode, effectively eliminating salt. This system has an ultra-high desalination capacity of 140.03 mg g^−1^ at a 20 mM salt concentration. Stable cycling performance exhibited maintained capacity throughout numerous cycles. The single-pass mode demonstrated the excellent desalination efficiency and durability of this dual-ion CDI system, indicating a potential method for future applications. Paroda *et al.*^75^ used activated carbon powder to create flow electrodes for continuous CDI and capacitive mixing energy generation. The AC had a surface area of 1450 m^2^ g^−1^ and a 1.01 nm pore size and was suspended in deionized water, homemade river water, or 0.25 M monoethanolamine (MEA) solution to boost CO_2_ solubility. Continuous operation was maintained at a flow rate of 2 ml min^−1^ for desalination and 1 ml min^−1^ for energy harvesting using an electrochemical potentiostat. Additionally, the increased carbon mass loading increased salt removal weight percent (wt%) by 20 wt%, while the extended cycle duration boosted efficiency. This mode of CDI allows single ion transfer, decreases energy losses and enhances performance. The CDI system, which used MnO_2_ as a cathode and NiO as the anode, achieved improved electrical conductivity and a specific adsorption capacity (SAC) of 21.01 mg g^−1^ in the pH range of brackish water. The oppositely^76^ charged surfaces formed by the dissimilar isoelectric points of MnO_2_ (pI = 4.5) and NiO (pI = 10) allowed for easier ion adsorption in single-pass CDI. Because of efficient electrosorption and intercalation, the system demonstrated high efficiency and stability over several charge–discharge cycles. Because single-pass CDI can operate continuously, it outperforms batch-made CDI and is therefore better suited for real-time desalination applications. It guarantees a constant water flow, minimizing downtime and increasing throughput in contrast to batch mode, which necessitates controlled cycles. This ongoing procedure enables more accurate control of desalination performance by real-time conductivity and salt removal monitoring. Furthermore, single-pass CDI removes the difficulties associated with recirculation, thereby lowering the maintenance needs and system design limitations. Additionally, it is an effective and scalable option for large-scale desalination requirements because it can deliver a steady supply of treated water.

## 4. Electrode materials for CDI

**Fig. 5:**
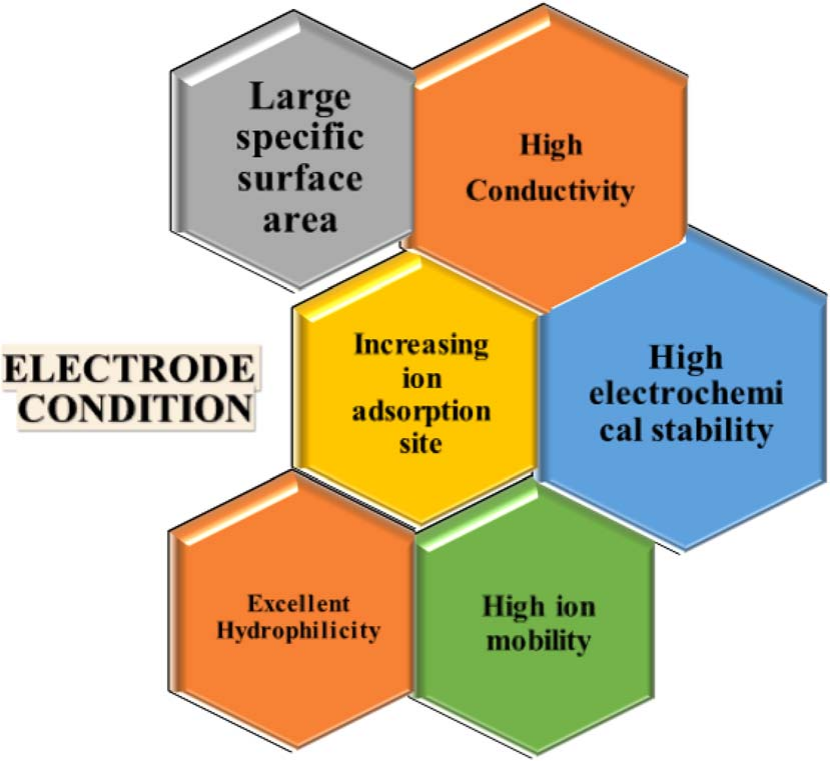
Schematic of conditions of the electrode materials.

**Fig. 6:**
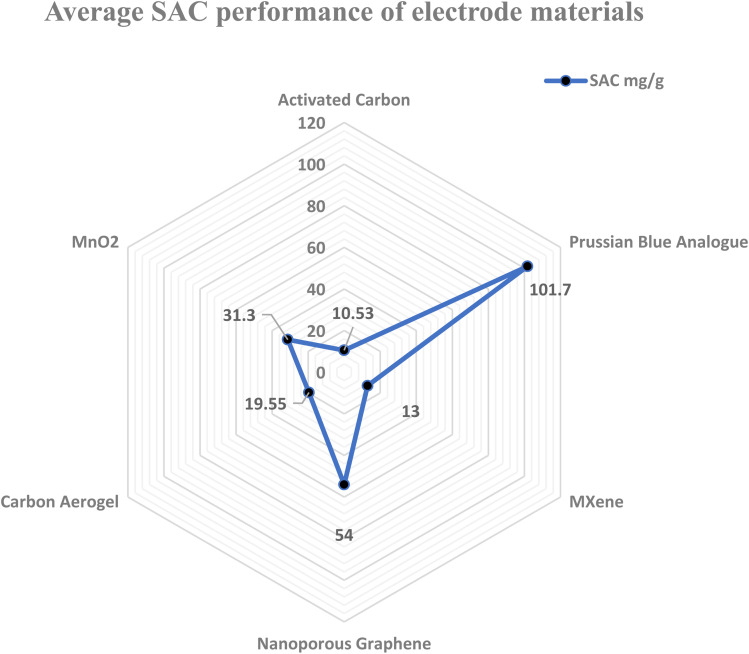
Web chart showing the average SAC performance of various electrode materials in capacitive desalination.

The physical and chemical characteristics of an electrode are important components for increasing the CDI efficiency. Therefore, various research teams have concentrated on developing electrode materials with high salt adsorption capacity (SAC) and an average salt adsorption rate (ASAR). Although porous electrodes with high surface areas improve the CDI performance, their SAC is limited by the absence of an ion intercalation process. The ideal electrode material for CDI depends on several variables, such as conductivity, surface area, chemical stability, and cost-effectiveness (Fig. 5). Thus, choosing high-performance electrode materials with certain qualities is essential for the development of CDI technology. Prussian Blue Analogues (PBAs), Transition Metal Oxides (TMOs), MXenes, Transition Metal Dichalcogenides (TMDs), and Metal–Organic Frameworks (MOFs) are examples of intercalation-based electrodes that show promise in addressing the drawbacks of conventional carbon electrodes^77^ and enhancing CDI efficiency and long-term survivability. Furthermore, the web chart analysis in Fig. 6 shows that in the research carried out over the previous ten years, PBA has shown the greatest SAC among the other materials.

Carbon-based materials such as activated carbon, carbon aerogels, and graphene with a large surface area may adsorb more ions through the EDLC process, improving desalination efficiency by the formation of an efficient electric double layer.^78–82^ Nevertheless, desalination capabilities can be further enhanced by employing ion intercalating materials, which store charge through an intercalation mechanism by insertion and extraction of ions within their structure.^83–85^ By employing both electrodes for CDI, the dual mechanism improves the overall desalination performance, increases the capacity for salt adsorption, and improves the efficiency of charge storage compared to carbon alone.^86–89^ Hence, non-carbon materials, such as transition metal oxides such as TiO_2_, Na_*x*_MnO_2_, V_2_O_5_, and MnO_2_, Prussian Blue Analogues such as FePO_4_, CoHCF, NiHCF (such as amorphous FePO_4_, NaTi_2_(PO_4_)_3_ and Na_2_V_2_(PO_4_)_3_, MXenes (such as Ti_3_C_2_T_*x*_)), and transitional metal dichalcogenides (such as MoS_2_, TiS_2_), have been explored as alternatives, which offer reversible ion storage mechanisms, improving SAC and ASAR in CDI applications.

### 4.1. Prussian blue analogues (PBAs)

PBAs have a high desalination capacity because of their open framework structure, which makes ion insertion and extraction more efficient. The high performance of high-entropy PBAs in CDI investigations can be attributed to several benefits. They are also inexpensive and eco-friendly, making them a good choice for large-scale water treatment projects and for successfully resolving water shortage problems worldwide. These materials have exceptional cyclic stability with over 97% capacity retention after 350 cycles because of the incorporation of high configurational entropy, which also improves structural stability and inhibits unfavourable phase transitions. The higher performance of high-entropy PBAs in CDI investigations can be attributed to numerous benefits. These materials have exceptional cycling stability with over 97% capacity retention after 350 cycles because of the incorporation of high configurational entropy, which also improves structural stability and inhibits unfavourable phase transitions. Lei *et al.*^90^ demonstrated that High Entropy PBAs (HE-PBAs) maintained their efficiency at varying salt concentrations and had a high desalination capacity of 77.24 mg g^−1^ at 1.2 V. Also, Na^+^ ion diffusion pathways were optimised due to the increased configurational entropy, which accelerated the reaction kinetics and increased efficiency. Additionally, to improve redox kinetics, which are necessary for efficient ion capture during the CDI process, Wang *et al.*^91^ in 2020 incorporated the networks of three-dimensional (3 D) carbon nanosheets with Nickel Hexacyanoferrate (NiHCF), formed a conductive framework that improved charge transfer kinetics, decreased resistance, and improved electron and ion transport. By preventing aggregation, the homogenous dispersion of NiHCF nanoparticles maximized the active surface area available for ion intercalation. In 2024, Li *et al.*^92^ introduced manganese (Mn) into the NiHCF structure. This decreased the energy barrier for ionic diffusion and increased the electronic conductivity. Furthermore, compared with conventional carbon electrodes, which frequently have poor cycling life, the hybrid structure of Mn–NiHCF coupled with polypyrrole (ppy) showed excellent cycling stability, sustaining performance over 50 cycles. A high charge efficiency of 81% was also demonstrated by optimised Mn–NiHCF/ppy, suggesting efficient use of active sites during desalination.^80^ PBAs have drawn a lot of interest for sodium ion capture applications by reducing the amount of [Fe(CN)_6_] vacancies.^93^ Their high crystallinity greatly increased their conductivity and promoted quick ion diffusion. A high SAC of up to 101.4 mg g^−1^ at 1.2 V was made possible by effective Na^+^ intercalation and deintercalation by the 3 D open framework of the PBAs. Furthermore, even after 100 cycles, the PBAs showed remarkable cyclic stability, continuing to function without noticeable degradation. The face-cantered cubic (FCC) shape of the Cobalt Hexacyanoferrate (CoHCF) layer offers a large number of redox-active sites, which makes it easier to intercalate and deintercalate Na^+^ ions during the HCDI process. To explain this synergistic effect, Ma *et al.*^94^ combined CoHCF with conductive polyaniline (PANI), which increased the specific capacitance of composite electrodes, accelerated ion transport, and improved the SAC (30.48 mg g^−1^) and ASAR (3.66 mg g^−1^ min^−1^). By combining conductive carbon nanofibers (CNFs) with PBAs, Wang *et al.*^95^ showed that conductive networks can be designed by improving electronic conductivity and electrochemical performance. In concentrated NaCl solutions, the PB/CNF combination demonstrated a remarkable SAC of 97.35 mg g^−1^, which also exhibited outstanding cyclic stability. Additionally, they were designed to be more hydrophilic, which further increased access to the salt solutions and made the active areas for ion adsorption more accessible.

### 4.2. Transition metal oxides (TMOs)

TMOs, including TiO_2_, MnO_2_, and V_2_O_5_, offer high redox activity and ion intercalation properties, making them promising candidates for CDI. However, their intrinsic low electrical conductivity necessitates the development of composites or conductive additives to improve their overall performance. In 2018, Santos *et al.*^96^ aimed to combine the hybrid architecture of porous CNFs with meta oxides to increase the effective charge storage capacity by optimizing the surface area. In their CDI investigations, they used SiO_2_ (silica) as the cathode and γ-Al_2_O_3_ (alumina) as the anode. According to their findings, brackish water (2.0 g NaCl/L) had a SAC of 6.5 mg g^−1^. They also showed a high charge efficiency of 86% and a low energy consumption of around 0.26 W h g^−1^ for salt removal. Subsequently, Yin *et al.*^97^ discussed the use of 3 D graphene/metal oxide nanoparticle hybrids, especially graphene aerogels combined with metal oxides such as TiO_2_, CeO_2_, Fe_2_O_3_, and Mn_3_O_4_, for enhanced desalination tests. These graphene/metal oxide hybrids demonstrated superior performance in CDI by achieving high electrosorption capacities, with the GA/TiO_2_ hybrids reaching a capacity of 25 mg g^−1^ for NaCl, significantly outperforming pure graphene aerogel and activated carbon. Additionally, the hybrids exhibited rapid electrosorption rates, achieving equilibrium under 200 seconds, and maintained excellent cyclic stability over 1000 cycles, underscoring their effectiveness as high-performance CDI electrode materials. By employing the MnO_2_/hierarchical porous carbon (HPC) composite in CDI, the study demonstrated remarkable results, exceeding previously reported values, with maximum SAC (mmol g^−1^) values of 0.65, 0.71 and 0.76 mmol g^−1^ for NaCl, MgCl_2_ and CaCl_2,_ respectively. Effective ion intercalation and improved desalination performance were made possible by the exceptional hydrophilicity, low charge transfer, and specific capacitance of 172.2 F g^−1^ of the MnO_2_/HPC composite.^98^ Additionally, due to greater binding interactions within the MnO_2_ structure, the composite demonstrated significant selectivity for divalent cations (Ca^2+^ and Mg^2+^) over monovalent cations (Na^+^), indicating its potential for water softening and brackish water desalination applications. In CDI applications, the V_2_O_5_ material showed notable advantages by achieving a SAC of 55.2 mg NaCl/g V_2_O_5_, indicating that salt ions were effectively removed. Fat ion diffusion and outstanding structural stability ensured the performance over several cycles. The strong conductivity and low charge transfer resistance were also emphasized in the study, which helped to reduce the energy consumption during desalination to 0.27 kW per h per kg-NaCl.^99^

### 4.3. MXenes

MXenes, such as Ti_3_C_2_T_*x*_, are 2D materials with excellent electrical conductivity and hydrophilic surfaces, which enhance ion transport in CDI. Despite these advantages, their SAC has been observed to be lower compared to PBAs, necessitating further optimization. Srimuk *et al.*^100^ used MXene (Ti_3_C_2_) as the anode and cathode in a CDI setup in an early investigation conducted in 2016. The material leveraged its unique intercalation mechanism, which enabled high charge storage capacity without requiring a large surface area. Both cations and anions could be intercalated effectively by their pseudocapacitance behavior, with an average salt adsorption capacity of 13 mg g^−1^ and high efficiency exceeding 97%. To improve the ion adsorption capacity, Li *et al.* introduced bacterial cellulose (BC) fibers to Ti_3_C_2_T_*x*_ MXene cathodes,^101^ which were able to efficiently control interlayer spacing by improving ion transport and active site exposure. With an enhanced charge efficiency that increased from 82.4% to 91.75%, this MXene/BC composite demonstrated strong hydrophilicity, which improved ion adsorption, high volumetric capacitance (∼1000 F cm^−3^), and outstanding conductivity (∼10 000 S cm^−1^). Furthermore, the MXene/BC-33% composite showed 66% thermodynamic energy efficiency (TEE), which was greater than that of pure MXene, suggesting increased desalination. Chen *et al.*^102^ combined MXenes (Nb_2_CT and Nb_2_O_5_) and reduced graphene oxide (rGO) for CDI applications in 2023. The dual-confinement of these composites improved the stability, inhibited Nb_2_O_5_ nanoparticle aggregation, and markedly increased the electrical conductivity. With an average adsorption rate of 4.14 mg g^−1^ min^−1^ and NaCl adsorption capacity of 41.07 mg g^−1^, this hybrid material showed exceptional desalination capability. Furthermore, it demonstrated outstanding long-term stability over 50 cycles with 81% desalination capacity retention. Compared to conventional materials, the dual-confinement structure produced more active sites for ion adsorption and accelerated ion diffusion, leading to better overall performance in CDI applications. Self-stacking was found to occur in MXenes, which hindered their electrochemical performance in all the previous studies; hence, Tan *et al.* in 2023 (ref. 103) employed MXenes with carbon dots to develop a hybrid composite, in which Ti_3_C_2_T_*x*_ MXene was employed as the cathode material in their investigation, and it was combined with carbon dots (MXene@CDs) in the shape of microflower, which has a low energy consumption of 0.13 kW per h per kg-NaCl, an energy recovery rate of 12.61%, and a high desalination capacity of 86.4 mg g^−1^ in 10 mM NaCl solution.

### 4.4. Transition metal dichalcogenides (TMDs)

TMDs, such as MoS_2_ and TiS_2_, possess layered structures that can accommodate ion intercalation. However, their low intrinsic conductivity presents challenges, and surface modifications or hybridization strategies are required to improve their performance. To develop miniature CDI devices for use in space-constrained contexts, the study by Han *et al.*^104^ showed that MoS_2_-Graphene hybrid electrodes had notable benefits when cast onto the graphite film, attaining a high gravimetric adsorption capacity of 19.4 mg g^−1^ and a high volumetric adsorption capacity of 14.3 mg cm^−3^. In 2021, Chai *et al.*^105^ successfully designed the MoS_2_@CNT-CS composite to provide an effective electrode material for treating brackish water at low concentrations. This innovative structure enhanced the MoS_2_ dispersion and provided a strong conductive foundation. Consequently, the electrode exhibited a particular ion selectivity order of Ca^2+^ > Na^+^ > Mg^2+^ > K^+^, with a noteworthy SAC of 25.35 mg g^−1^ and a quick SAR of 3.9 mg g^−1^ min^−1^. To gain knowledge of sodium ion intercalation into material interlayers at the atomic level, Li *et al.* successfully clarified the ion intercalation process in CDI using TiS_2_.^106^ This work methodically examined the stacking patterns, intercalation locations, and energetics of intercalated compounds using Density Functional Theory (DFT) simulations. The proposed strategy confirmed the computational techniques and provided insightful information about phase changes during sodium intercalation. Given their encouraging intercalation characteristics, TMDs have emerged as viable options for improving desalination efficiency in CDI applications. Their distinct layered structures provide effective ion insertion, which enhances their salt adsorption capacity. To improve stability, conductivity, and overall CDI performance, future studies should focus on optimizing TMD-based electrodes through structural changes and surface functionalization. Moving this way can open a new path for the development of desalination systems with great efficiency.

### 4.5. Metal–organic frameworks (MOFs)

MOFs provide tunable porosity and high surface area, making them potential candidates for CDI applications. Their unique structures enable selective ion adsorption, but stability and conductivity enhancements are necessary for practical implementation. To develop a new family for CDI electrodes, Wang *et al.*^107^ introduced MOFs with polypyrrole, which combines high electrical conductivity and a high specific surface area (SSA) of 1176.8 m^2^ g^−1^, whereas this hybrid enabled efficient electron transfer and salt adsorption of 11.34 mg g^−1^. Further studies were conducted in comparison of N-doped carbon particles (NCPs) and AC with N-doped carbon tubes derived from MOFs as CDI electrode materials. Tubular construction that decreased diffusion distances, binder-free arrangement that increased accessible surface area, and nitrogen doping improved the reactivity and conductivity of NCTs, as reported by Xu *et al.* in 2020.^108^ These characteristics allowed NCTs to overcome NCPs and Acs by achieving a SAC of 56.9 mg g^−1^. Furthermore, ion transport and adsorption were improved by the logical design and synthesis of the hollow carbon structure, thereby proving its usefulness in desalination technologies. The incorporation of multi-heteroatom co-doping allowed for much greater improvement by boosting the electrochemical characteristics of the carbon material. Using a graphite sheet as the current collector, Zhang *et al.*^109^ expanded on this strategy by using N, P, and S co-doped hollow carbon polyhedrons derived from MOF-based core–shell nanocomposites (ZIF-8@PZS-C) as the anode material in CDI. Superior ion adsorption and transport were facilitated by the SSA of the anode material, which increased further hydrophilicity, higher electrical conductivity and decreased internal impedance. Consequently, in 500 mg per L NaCl solution, the ZIF-8@PZS-C electrodes exhibited an SAC of 22.19 mg g^−1^ at 1.2 V.

We systematically evaluated various electrode materials such as Prussian Blue Analogues (PBA), transitional metal oxides (TMOs), transitional metal-dichalcogenides (TMDs), MXenes and metal organic frameworks (MOFs) for their effectiveness in CDI. Our comparative analysis reveals that every class of materials has unique advantages as well as limitations with regard to long-term stability, charge-storage approaches, and SAC. MXenes show high SAC and charge efficiency due to their superior ion transport capabilities, high conductivity and hydrophilicity. In contrast, PBAs have proven popular for hybrid CDI applications because of their quick ion intercalation kinetics and excellent desalination performance. Despite providing redox-driven charge storage, TMOs exhibit poor electrical conductivity, further reducing their overall CDI efficiency. However, the use of optimized TMOs and PBAs in hybrid CDI enhances desalination performance by utilizing EDLC and faradaic charge storage mechanisms.

## 5. Synthesis strategies for ion intercalation materials

To enhance CDI performance with Prussian Blue Analogues (PBAs), an innovative material in the field of desalination, exhibiting a unique open 3 D framework and efficient ion exchange capabilities. A comprehensive approach encompassing the synthesis, characterization, and electrode design is crucial. The precise selection of transition metal ions (*e.g.*, Fe, Co) of PBAs and bridging cyanide ligands to tailor redox potential and structural stability, impacting ion affinity and transport. The synthesis method includes electrochemical oxidation using hydrothermal,^110^ co-precipitation^111^ electrodeposition^112^ sol gel,^113^ and many other routes, which significantly influence morphology, particle size,^114^ crystallinity,^115^ and pore size,^85^ allowing strategic manipulation of PBA properties. The structure of PBAs allows for the selective exchange of sodium ions (Na^+^) with larger divalent ions like magnesium (Mg^2+^) and calcium (Ca^2+^) from seawater or brine. This selective ion removal not only enhances desalination performance and contributes to the rapid diffusion of ions and water through their porous structure, ensuring high ionic conductivity and efficient ion transport.

### 5.1. Hydrothermal

Using a straightforward hydrothermal technique, Yang *et al.* synthesised a MXene VO_2_/CTAB-Ti_3_C_2_ heterostructure that effectively inhibited the re-stacking of Ti_3_C_2_ layers.^116^ It exhibited a distinctive lamellar morphology that improved the surface roughness and interlayer spacing, with an SSA of 28.06 m^2^ g^−1^. This structure outperformed CTAB-Ti_3_C_2_ (4.36 m^2^ g^−1^) and was lower than pure VO_2_ (33.77 m^2^ g^−1^). Another study focused on plasma-treated vanadium-based material (NH_4_)_2_V_10_O_25_·8H_2_O (P-NVO), synthesized *via* the hydrothermal method,^117^ further enhancing its properties by argon plasma treatment. P-NVO exhibited a micro flower-like morphology with a layered crystalline structure and 0.97 nm interlayer spacing, retaining its integrity post-treatment. This morphology increased the surface area for ion adsorption and facilitated Pb^2+^ intercalation, enabling efficient ion transport and storage. The hierarchical nanoneedles improved electrode–electrolyte contact, reducing resistance and enhancing ion diffusion. Plasma treatment introduced oxygen vacancies, creating additional active sites and P-NVO demonstrated a specific capacitance of 204.6 F g^−1^ at 1 A g^−1^ and a high adsorption capacity of 49.56 mg g^−1^ for Pb^2+^ from a 400 mg per L Pb(NO_3_)_2_ solution, showing superior selectivity for Pb^2+^ over Cd^2+^ compared to traditional carbon electrodes. To enhance the performance of salt adsorption capacity, Tan *et al.* synthesized a hybrid electrode material composed of Ti_3_C_2_T_*x*_, MXene and carbon dots (CDs), referred to as MXene@CDs, using a hydrothermal method for CD synthesis and electrostatic self-assembly for composite film formation. The hybrid material exhibited a unique microflower structure that minimized the self-stacking of MXene nanosheets, improving the ion transport and storage capacity for capacitive deionization (CDI) applications.^103^ The morphology featured interspersed MXene nanosheets and CDs, resulting in a flexible, binder-free electrode with a high specific surface area that facilitated enhanced ion diffusion and adsorption. CDI studies showed that the MXene@CDs-2 electrode achieved a high desalination capacity of 86.4 mg g^−1^ at a current density of 20 mA g^−1^, with low energy consumption of 0.13 kW per h per kg-NaCl and an energy recovery rate of 12.61%. These results highlighted the significant performance enhancement due to the hybrid structure and demonstrated its potential for efficient water desalination. In 2023, MnO_2_/PANI composites were hydrothermally synthesized after hierarchical porous carbon (HPC) was been synthesized from microalgae using a one-pot pyrolysis technique.^98^ The MnO_2_/HPC composite showed a multimodal pore size distribution and a large surface area of approximately 175.2 m^2^ g^−1^, with MnO_2_ nanorods uniformly anchored onto the HPC surface. This special morphology and shape promote ion accessibility, buffer volume fluctuations during ion intercalation and deintercalation, and increase electrical conductivity. Furthermore, in advance to research on PBA, Vafakhah *et al.*^118^ synthesized sodium vanadium fluorophosphate wrapped in reduced graphene oxide (NVPF@rGO) using the hydrothermal method by mixing precursors in deionized water and heating in a Teflon-lined autoclave. The material exhibited micro-sized (∼3 μm) and nano-sized (<600 nm) morphologies, with the latter providing enhanced electroconductivity and faster ion migration. In capacitive deionization (CDI), the nano-sized particles achieved a high salt adsorption capacity (60 mg g^−1^) and 98% retention over 500 cycles. These results highlight NVPF@rGO as a promising electrode material for efficient and stable desalination applications.

### 5.2. Co-precipitation

**Fig. 7:**
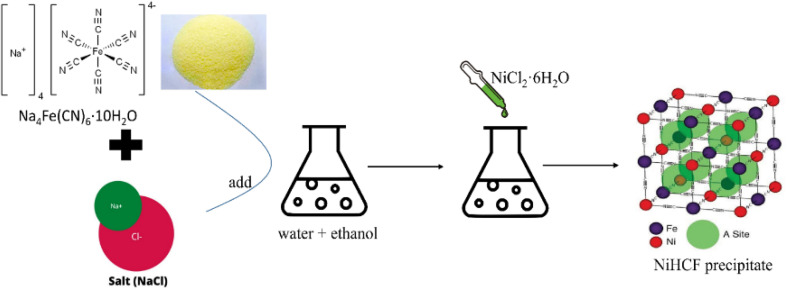
Schematic of synthesis of nickel hexacyanoferrate.

To enhance charge transfer, high active sites, and reduced ion diffusion, Xu *et al.*^89^ synthesized Nickel hexacyanoferrate with a carbon nanotube (NiHCF/CNT) composite *via* a co-precipitation technique that involved acidifying CNTs with sulphuric and nitric acids. The final product had homogenous 300 nm NiHCF particles interconnected by a 3 D network of carbon nanotubes, which further enhanced charge transfer and inhibited NiHCF agglomeration due to its high surface area of 205 m^2^ g^−1^ and achieved a SAC of 29.1 mg g^−1^. Furthermore, in 2017, Slawomir Porada *et al.*^119^ achieved continuous desalination demonstration within a CDI cell for the first time experimentally and was carried out by intercalating Na^+^ ions from a NaCl solution using redox-active NiHCF nanoparticles, which were synthesized by a solution/precipitation reaction with aqueous reagents using the following synthesis reaction:

NiCl_2_ + Na_4_Fe(CN)_6_ → Na_2_NiFe(CN)_6_ + 2NaCl and characterized the dynamic, cyclic behaviour of the CDI cell at a salinity typical of brackish water. The general synthesis method is shown in Fig. 7, which shows the synthesis of NiHCF using Na_4_Fe (CN)_6_ by Lee *et al.*^120^ in a controlled crystallization reaction with citrate. These results show that the system has a high desalination capacity (59.9 mg g^−1^) and efficient energy consumption (0.34 W h L^−1^ for 40% Na ion removal efficiency). PBAs typically exhibit structural flaws and poor electronic conductivity, resulting in weak cycle stability and reduced capacity. To overcome these drawbacks, Ao Gong *et al.*^121^ incorporated iron-based Prussian blue (FeHCF) in a three-dimensional nitrogen-doped carbon framework (3 DNC). The FeHCF@3DNC electrode showed a high desalination capacity of 60.5 mg g^−1^ and excellent cycle repeatability.

### 5.3. Electrochemical method

**Fig. 8:**
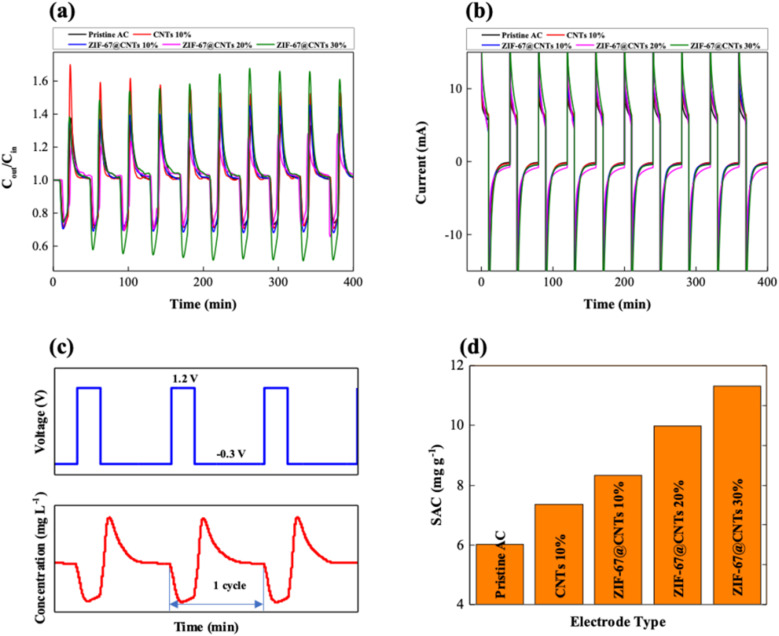
Variation in the (a) salt concentration of effluent stream and (b) measured current during CDI desalination for 10 cycles. (c) Potential difference applied to the CDI cell (top) and change in effluent concentration magnified from (a) (bottom). (d) Calculated salt adsorption capacity of various CDI electrodes.^124^

Research on PBA materials has highlighted the promising potential of various PBA morphologies. In 2010, Sabzi *et al.*^122^ demonstrated a method for synthesizing nickel hexacyanoferrate (NiHCF) *via* electrochemical oxidation of nickel in the presence of hexacyanoferrate ions and a nanoporous anodic alumina oxide (AAO) template. This study successfully produced NiHCF nanoarrays with distinct morphologies—nanodots (70–90 nm), nanorods (2–5 μm length, 70–90 nm diameter), and nanotubes (30–50 nm diameter). The electrochemical analysis revealed that NiHCF nanorod arrays exhibited superior current responses and electrochemical performance compared to other morphologies, with their larger surface area (50–100 m^2^ g^−1^) and reduced diffusion limitations. Furthermore, the CDI studies of NiHCF nanorods showed high specific adsorption capacity (200–300 mg g^−1^), with Na^+^ ion removal efficiency (up to 90%), and the effective diffusion coefficient was reported as 1.2 × 10^−6^ cm^2^ s^−1^, with peak currents reaching 1.5 mA. Furthermore, Alberto Martinez *et al.*^123^ investigated the electrochemical synthesis of some metal–organic frameworks, such as HKUST-1, ZIF-8, MIL-100(Al), MIL-53(Al), and NH_2_-MIL-53(Al). This synthesis method has several advantages over traditional hydrothermal methods, including faster synthesis times, milder reaction conditions, and the ability to synthesize MOF nanoparticles with tunable morphologies. The critical synthesis parameters, including solvent, electrolyte, current density, and temperature, significantly affect the yield, crystallinity, and textural characteristics of the final MOFs. Interestingly, the electrochemically synthesized MIL-53 and NH_2_-MIL-53 samples exhibit less framework flexibility compared to their hydrothermally synthesized counterparts, which is most probably due to the fast formation kinetics. This would allow the construction of MOF coatings on conductive surfaces, such as demonstrated by the example with HKUST-1which was been synthesised by electrochemical deposition. Alberto Martinez *et al.* investigated the electrochemical synthesis of some metal–organic frameworks, such as HKUST-1, ZIF-8, MIL-100 (Al), MIL-53 (Al), and NH_2_-MIL-53. Ngo Minh Phuoc *et al.*^124^ in 2020, synthesised ZIF-67 and combined with CNT, which illustrates the investigation of the synthesized ZIF-67@CNT, which enhanced the CDI system. The CDI cell was initially charged for 10 min at 1.2 V and then discharged for 30 min at −0.3 V, and this process was repeated 10 times (Fig. 8c). The electro-adsorption of Na^+^ and Cl^−^ ions onto the electrode surfaces during charging decreased the concentration of the effluent salt, which subsequently increased as electrode saturation took place. The desorbed ions caused the effluent concentration to rise above the influent level during the discharge period (Fig. 8a and b). The SAC of different CDI electrodes is displayed in Fig. 8d, which shows that pristine AC has the lowest SAC (6.01 mg g^−1^). With 10 wt% CNT, the SAC increased to 7.35 mg g^−1^, and with 10, 20, and 30 wt% ZIF-67@CNT, it increased to 8.32, 9.98, and 11.32 mg g^−1^, indicating improvements of 38%, 66%, and 88%, respectively. The combined advantages of the porosity and increased conductivity of ZIF-67 and CNT are reflected in this improvement.

### 5.4. Chemical vapor deposition (CVD)

Further studies on V_2_O_5_ using chemical vapor deposition (CVD) at 625 °C significantly enhanced the material's electrical conductivity and resulted in a honeycomb structure that provided a high surface area essential for effective ion adsorption during CDI, while the graphene coating improved charge transfer and the morphology remained structurally intact throughout multiple desalination cycles, ensuring stable performance without significant degradation. These findings demonstrated the synergistic benefits of the unique morphology in enhancing electrode performance for CDI applications.

### 5.5. Calcination/etching

The N, P, and S co-doped hollow carbon polyhedron was been synthesised by using the ZIF-8 template, forming a ZIF-8@PZS composite by calcining/etching.^109^ The material had a hollow polyhedron structure of 70 nm with a shell of 8 nm, a surface area of 929 m^2^ g^−1^, and a pore volume of 1.60 cm^3^ g^−1^. By improving ion transport, its mesostructured material achieved a high SAC of 22.19 mg g^−1^ in 500 mg per L NaCl at 1.2 V. Co-doping enhances hydrophilicity and conductivity, thereby increasing the CDI performance.

### 5.6. Other synthesis routes

Additionally, synthesis parameters such as temperature, pH, and reactant concentrations are crucial, and hence, in 2021, Arulrajan *et al.* highlighted how pH (ref. 125) variations are influenced by feed water type and electrode condition. They observed that the pH decreased during the desorption of NaCl solution, whereas tap water containing carbonate and bi-carbonate ions increased the pH. The preferential adsorption of bicarbonate ions during desalination contributed to pH fluctuations, raising concerns about scaling risks at higher recoveries Lumley *et al.*^126^ demonstrated the structure–composition–property relationships of nickel hexacyanoferrate (NiHCF) by explaining how synthesis conditions, such as temperature and precursor selection, affected the crystallinity, morphology, and electrochemical performance of NiHCF. The redox reactions of Fe ions (Fe^2+^/Fe^3+^) during sodiation and desodiation were coupled with the insertion and release of Na^+^ ions within the NiHCF framework. This study emphasized the influence of the lattice type (cubic *vs.* rhombohedral), A-site occupancy, and Fe(CN)_6_ vacancies on the electrochemical behavior and stability. By analyzing these factors, the authors identified ways to optimize NiHCF for improved performance in capacitive deionization (CDI). The cubic lattice structure with fewer vacancies exhibited higher stability and enhanced Na^+^ storage capacity. These findings contribute to the development of efficient NiHCF-based materials for practical seawater desalination and energy storage applications. Further improvement was made to develop a promising redox-active intercalation electrode material for hybrid capacitive deionization (HCDI) by synthesizing NiHCF@3DC-2, a redox-active intercalation electrode combining PBA nanoparticles with a 3D carbon nanosheet network. The synthesis involves a two-step process: forming a SiO_2_-coated citric acid precursor, freeze-drying, and carbonizing under argon to create a porous 3D carbon framework, followed by the *in situ* growth of NiHCF nanoparticles (10–20 nm) within macropores (0.8–1 μm). This structure enhances ion transport and electron transfer and prevents aggregation, yielding a high ion removal capacity of 107.5 mg g^−1^ in 20 000 mg per L NaCl with a peak ion removal rate of 11.0 mg g^−1^ min^−1^. The material's conductivity, porosity, selective cation intercalation, and cycling stability (90.3% retention over 120 cycles) contributed to its superior performance. To maximize ion transport, Ahn *et al.* in 2024 (ref. 127) combined cobalt chloride, sodium citrate, and sodium hexacyanoferrate and stirred the mixture for 24 h, then aged and filtered, NaCoHCF electrodes were synthesized using the citrate-assisted controlled crystallization technique. The resultant NaCoHCF had larger dimensions than usual PBA particles, measuring about 5 μm, and a highly crystalline face-centered cubic structure. High desalination rates were achieved, which was made possible by this structure with improved ion transport during CDI and an increased specific capacity of 88 mA h g^−1^, which further increased effective ion intercalation and deintercalation. Further research focused on MXenes and their composites, in which Taha *et al.* synthesized titanium oxynitride (Ti_*x*_O_*y*_N_*z*_) nanoparticles *via* nitridation and annealing TiO_2_ in an ammonia atmosphere. This process produced a *meso*-macroporous structure (∼23 nm pore diameter), enhancing the capacitive deionization (CDI) performance.^128^ The nanoparticles achieved a high salt adsorption capacity (SAC) of 56.6 mg g^−1^ and retained nearly 100% SAC after 1960 cycles over 110 days. The interconnected porous structure facilitates rapid ion transport and improved electrode-ion interactions. Compared to conventional carbon-based electrodes with lower SAC (∼15 mg g^−1^) and stability issues, transitional metal oxides such as MnO_2_, TiO_2_, RuO_2_, Fe_2_O_3_/Fe_3_O_4_ are integral to the advancement of CDI technologies, offering pathways to more efficient and sustainable water desalination solutions by removing ions by electrochemical methods. The unique properties of TMOs are their high specific capacitance and pseudocapacitive behaviour, making them excellent candidates for the CDI electrode. It can also show both Electrical Double Layer Capacitance (EDLC) or pseudocapacitance (Ion Intercalation mechanism), but most of the TMOs primarily operate through pseudocapacitance, *i.e.*, it stores charge *via* surface or near-surface redox reactions, involving reversible faradaic reactions due to the intercalation/deintercalation of cations (Na^+^ or K^+^) in its lattice. TMOs such as PBA operate through reversible redox reactions, enabling efficient ion intercalation and charge storage, but other TMOs, such as MnO_2_ and TiO_2_, are synthesized using techniques like thermal decomposition and chemical precipitation, producing materials with tailored crystal structures and conductivity.^129^ The combination of high capacity, structural stability, and redox activity of TMOs makes them particularly effective in flow-between CDI systems, where energy efficiency and cycling stability are critical. These synthesizing strategies highlight the potential of TMOs, especially PBAs, to advance CDI technologies by improving ion capture efficiency and overall performance.^130^ Manganese oxide (MnO_2_)^88,131^ and Ruthenium dioxide (RuO_2_)^132^ exhibit high specific capacitance due to their ability to undergo reversible redox reactions, enhancing the capacity of CDI applications. The behavioural mechanism also depends on which electrolyte is used for analysis, such as alkaline, neutral and acidic electrolytes. Although the material is the same, the CV behaviour will be different with different electrolytes. MnO_2_ exhibits E DLC behaviour in a neutral medium because of its stable environment and prevents extensive redox reactions, limiting the material's response to surface adsorption, whereas, in an alkaline medium, MnO_2_ undergoes reversible faradaic reactions of OH^−^, which interact actively with electrode materials. The major advantage lies in engineering the TMOs into various nanostructures to prevent electrode stacking and improve electronic conductivity, thereby enhancing CDI performance. To introduce a new family to the electrode materials of CDI, MXenes emerged as the most promising materials for CDI due to their high electrical conductivity and large surface area. However, it tends to restack, which may degrade the electrochemical performance. Kajiyama *et al.*^84^ explained that the Ti_3_C_2_T_*x*_ electrode, which was been synthesised using a simple precursor method, shows reversible behaviour because the layered structure allows sodium ions to access and depart during the sodiation/desodiation process. During the initial sodiation, a significant expansion of the interlayer spacing (from 9.7 Å 12.5 Å) is observed due to the insertion of desolvated sodium ions and the penetration of solvent molecules. This expansion is stabilized by the trapped sodium ions and the swelling effect of the solvent, which helps to maintain the increased interlayer distance even in subsequent cycles. Furthermore, faradaic charge storage was established by combining VO_2_, a redox-active material, with highly conductive Ti_3_C_2_–MXenes, improving ion capture above and beyond traditional double-layer adsorption.^116^ The remarkable desalination performance of NVPF@rGO was facilitated by the creation of a porous conductive network by reduced graphene oxide (rGO), which enhanced charge transfer, preserved electrode integrity, and allowed for rapid Na^+^ intercalation/deintercalation. Although the hierarchical pore structures in both composites offered numerous active sites for ion adsorption, the pseudocapacitive behavior of vanadium in NVPF further enhanced charge storage. Furthermore, their exceptional structural stability guaranteed little degradation; over 500 cycles, NVPF@rGO retained 98% SAC, and over several desalination cycles, VO_2_/CTAB-Ti_3_C_2_ demonstrated significant SAC retention. These materials were incredibly effective for CDI due to their balance of high electrical conductivity, large surface area, optimized pore architecture, and enhanced electrochemical stability. This enables long cycling performance, high adsorption efficiency, and quick ion transport for next-generation CDI applications. Whereas, Metal–Organic Frameworks (MOFs) are emerging as advanced materials for CDI due to their highly customizable structures, high porosity, large surface area, and unsaturated metal centre pores with high thermal and chemical stability and exceptional selectivity.^133^ MOFs are synthesized through methods like sonochemical synthesis^134^ and microwave-assisted synthesis, which allow precise control over crystal size, morphology, and pore structure.^135^ These techniques ensure that MOFs exhibit the structural stability and functional tunability required for efficient CDI performance. The unique framework of MOFs, which comprises metal ions and organic linkers, allows selective ion trapping and enhanced adsorption capacity. Their customizable pore sizes allow for precise ion sieving, reducing energy consumption and improving desalination efficiency. MOFs are particularly effective in hybrid CDI systems, where their selective ion capture capabilities complement other materials like graphene. The ability to fine-tune their chemical and structural properties through synthesis makes MOFs a promising material for advancing CDI technologies.^136,137^ The potential to achieve high performance with minimal energy input positions them as a key component in next-generation desalination solutions. The study by Wang *et al.*^107^ focused on a hybrid material composed of polypyrrole (PPy) nanotubes and a MOF, specifically, a Zeolitic Imidazolate Framework-67 (ZIF-67). When a 2-methylimidazole (2-MeIM) methanolic solution is added, the pre-synthesized PPy nanotubes are dispersed in a Co(NO_3_)_2_ methanolic solution, where they adsorb free Co^2+^ ions and act as nucleation sites for the development of ZIF-67 crystals. With well-dispersed ZIF-67 polyhedral interlaced with PPy nanotubes and an average diameter of 230 ± 78 nm for PPy nanotubes and an average size of 1.49 ± 0.28 μm for the ZIF-67 particles, the resulting ZIF-67/PPy hybrid displays an interconnected network topology. By lowering the overall bulk electrical resistance, promoting quicker electron transfer, and producing a high desalination capacity (SAC) of 11.34 mg g^−1^—much higher than the capacities of pure ZIF-67 (3.82 mg g^−1^) and PPy nanotubes (6.92 mg g^−1^), this distinctive morphology improves the hybrid's electrical conductivity. This hybrid is a potential option for capacitive deionization (CDI) applications because of the high porosity of ZIF-67 and the strong electrical conductivity of PPy.

The various synthesis methods for ion intercalation materials in CDI are hydrothermal, co-precipitation, electrochemical synthesis, chemical vapor deposition (CVD) and microwave-assisted synthesis. Materials synthesized using the hydrothermal method provide better control over crystal structure, defect engineering, and phase purity, which are essential for increasing ion intercalation capacity. Precise morphological control is made possible by hydrothermal synthesis, which affords distinct crystalline structures that promote effective charge storage and ion transport. It also encourages the development of porous structures and large surface areas, which enhance ion mobility and electrolyte accessibility. In addition, it can provide superior structural and electrochemical properties, maximize SAC, enhance charge storage kinetics, and lower energy consumption in CDI applications which has been represented in Table 1. Hydrothermal synthesis is generally the best method for developing high-performance ion intercalation materials. Furthermore, co-precipitation is more economical and scalable than other methods, although it frequently produces materials with less crystallinity and uneven phase control. Rapid heating is made possible by microwave-assisted synthesis, which improves phase purity and defect generation although more refinement is required to achieve uniform particle size (Table 1).

**Table 1:** Performance of various electrode materials in capacitive deionization

Sl no.	Materials	Feed concentrations	Flow rate (ml min^−1^)	Voltage (V)	SAC (mg g^−1^) ref.
1	NiHCF/C	500 mg L^−1^	100 ml min^−1^	1.2 V	29.1 mg g^−1^ (ref. 89)
2	Rhombohedral-NiHCF	0.6 M NaCl		1.4 V	126
3	CoHCF@PEDOT (HCDI)	1 mol L^−1^ NaCl		1.2 V	146.2 mg g^−1^ (ref. 138)
4	CuHCF (FT & FB-CDI)	50 mM NaCl	0.25 ml min^−1^	1.2 V	19 mg g^−1^ (ref. 55)
5	FeFe (CN)_6_ (PB)	500 to 10 000 ppm NaCl	50 ml min^−1^ to 950 ml min^−1^	1.2 V	120 mg g^−1^ (ref. 44)
6	(NH_4_)_2_V_10_O_25_·8H_2_O (NVO)//AC (HCDI)	1 M NaCl	10 ml min^−1^	1.2 V	49.56 mg g^−1^ (ref. 117)
7	MXene@CDs-2	10 mM NaCl	7 ml min^−1^	1.2 V	86.4 mg g^−1^ (ref. 103)
8	NTP-MXene/rGO	10 mM NaCl	50 ml min^−1^	1.8 V	251.55 mg g^−1^ (ref. 139)
9	Ti_3_C_2_-MXene (FBCDI)	5 mM NaCl	22 ml min^−1^	1.2 V	13 mg g^−1^ (ref. 100)
10	Na_3_V_2_(PO_4_)_3_@Carbon	1000 mg L^−1^ NaCl	20 ml min^−1^	1.2 V	74.0 mg g^−1^ (ref. 140)
11	Co_3_V_2_O_8_@C	500 mg L^−1^ NaCl	40 ml min^−1^	1.2 V	160 mg g^−1^ (ref. 141)
12	FeNC	100 ml NaCl	10 ml min^−1^	1.2 V	28.88 mg g^−1^ (ref. 142)
13	Nb_2_O_5_/Nb_2_CT_*x*_-Rgo (HCDI)	1 M NaCl		1.2 V	41.07 mg g^−1^ (ref. 102)
14	MnO_2_/RGO//RGO (HCDI)	500 mg L^−1^	10 ml min^−1^	1.2 V	52 mg g^−1^ (ref. 143)
15	Ti_*x*_O_*y*_N_*z*_ (flow-by CDI)	350 mg L^−1^	30 ml min^−1^	1.2 V	56.6 mg g^−1^ (ref. 128)
16	NVPF@rGO (flow-by CDI)	5000 ppm NaCl	50 ml min^−1^	1.2 V	60 mg g^−1^ (ref. 118)
17	G-V_2_O_5_ (flow-by CDI)	5 mM NaCl	40 ml min^−1^	1.4 V	6.7 mg g^−1^
18	N-doped carbon quantum dots/vanadium nitride (NCQDs/VN)	10 mM NaCl	40 ml min^−1^	1.2 V	8.5 mg g^−1^
19	TiO_2_@CNF (FT-CDI)	15 mM NaCl	5 ml min^−1^ to 40 ml min^−1^	1.4V	12.5 mg g^−1^ (ref. 144)
20	N-doped hollow mesoporous carbon sphere and holey graphene hydrogel (N-HMCS/HGH)	500 mg L^−1^	25 ml min^−1^	1.4 V	23.71 mg g^−1^ (ref. 145)

## 6. Conclusion

A promising approach for desalinating water and removing impurities is capacitive desalination (CDI). The efficiency of CDI systems hinges on ion intercalation, which governs electrode performance and efficiency. The ongoing research aims to enhance electrode materials for improved intercalation, thereby reducing costs and enhancing energy efficiency in CDI systems. In comparison with conventional desalination methods like reverse osmosis (RO), which typically consume 1–3 kW h m^−3^ for freshwater production, CDI offers low energy consumption rates ranging from 0.5 to 1 kW h m^−3^. However, CDI is not devoid of fouling and scaling issues. Identifying the optimal electrode materials remains a challenge despite the mature state of CDI technology. Novel electrode materials capable of faradaic reactions, surface redox reactions, and improved ion electrosorption have been explored to enhance CDI performance. Many distinct ion intercalation materials have been used with different cell topologies, and high SAC has been demonstrated. Much research has been conducted on carbonaceous electrodes because of their large surface area and porosity, which are useful in capacitive desalination. Various electrode materials, including carbon-based, non-carbon-based, and ion intercalation materials, play crucial roles in CDI systems, each with its own set of advantages and drawbacks. With their superior electrical conductivity, carbon-based materials like graphene and activated carbon allow for rapid ion adsorption and desorption during CDI. These materials are cost-effective, readily available and exhibit superior electrical conductivity, making them suitable for large-scale CDI applications. In particular, activated carbon can provide a high surface area for ion adsorption, thereby maximizing the CDI system efficiency. However, these materials typically have lower specific ion storage capacities than intercalation materials and may necessitate larger electrode volumes, potentially leading to bulkier CDI systems. Some carbon materials can even exhibit irreversible ion adsorption, reducing the CDI system's efficiency and lifespan.

In contrast, ion intercalation materials, such as Prussian blue analogues, transitional metal oxides and meta organic frameworks, offer high specific capacities for ion storage and tunability through compositional modifications. Many intercalation materials exhibit excellent ion insertion/extraction reversibility, which is crucial for the long-term stability and efficiency of CDI systems. We can compare a few electrode types in this review to gain a better understanding of their usefulness in CDI applications. The selection of ion intercalation materials or carbon-based materials as CDI electrodes is based on specific application requirements. Compared with carbon-based materials, ion intercalation materials offer superior long-term stability, tunability, and large ion storage capacity. The synergistic interaction between the pseudocapacitive features of intercalation materials and the EDL formation of carbon-based materials has demonstrated promising results for performance enhancement when intercalation materials and carbon-based materials are combined. While certain intercalation-based systems have demonstrated excellent desalination capabilities, most have limited ability to remove anions such as chloride ions. In reality, a hybrid strategy that utilizes the advantages of both electrode types—using carbon-based materials for effective ion kinetics and ion intercalation materials for high-capacity storage—has provided the greatest approach. The design of CDI systems requires careful consideration of these factors to effectively meet the desired performance and cost objectives. Researchers and engineers must continue to explore and innovate electrode materials to further advance the capabilities and efficiency of CDI technology.

To increase the desalination effectiveness of ion-intercalating materials like PBA, structural alterations must be made through composite synthesis. PBA-based composites offer a distinct advantage over traditional carbon-based materials by boosting ion transport kinetics and increasing charge storage capacity. It is possible to design a crystal lattice with enlarged interstitial gaps using controlled synthesis techniques that accelerate ion intercalation and prevent structural deterioration. These changes resulted in a much higher SAC by optimizing the electrochemical performance and mitigating lattice flaws. In addition, improving the control of surface chemistry, porosity, and crystallinity by synthesis parameter adjustment leads to improved adsorption kinetics and ion accessibility. In comparison with carbon-based electrodes, the synergistic effect of PBA and appropriate composite materials resulted in enhanced conductivity, mechanical stability, and greater desalination efficiency. PBA composites are a viable option for next-generation CDI technologies because they can offer improved energy efficiency and longer electrode lifespans by incorporating structural tuning techniques. This study demonstrates the superiority of composite engineering in improving CDI performance and opening the door to the creation of desalination systems with larger capabilities that are robust and scalable.

## 7. Future perspectives

Investigating Prussian Blue Analogue (PBA)-based CDI architectures that operate without anion exchange membranes is crucial because, despite their impressive desalination capabilities, which range from 20 to 200 mg g^−1^, PBAs' dependence on AEM raises system costs. The successful desalination of ultra-low salinity water (<100 ppm), a major obstacle for next-generation water purification technologies, is one of the crucial issues that future developments in CDI must address. As the field develops, scientists should consider the complex relationship between the vacancy distribution and physical characteristics of PBAs. They can even implement state-of-the-art methods such as electron diffraction and serial femtosecond crystallography to learn more about structural dynamics. Further improving the ion adsorption efficiency may be possible by precisely controlling the vacancy networks through knowledge of the effects of cooperative Jahn–Teller distortions and alkaline–metal inclusion. To address the difficulties of water shortage, these paths provide fascinating prospects for present-day young researchers to develop CDI systems with enhanced selectivity, reduced energy use, and superior performance.

## Conflicts of interest

The authors declare that they have no known competing financial interests or personal relationships that could have influenced the work reported in this paper.

## Data Availability

No primary research results, software or code has been included and no new data were generated or analysed as part of this review.

## References

[cit1] Luciano M. A., Ribeiro H., Bruch G. E., Silva G. G. (2020). Efficiency of capacitive deionization using carbon materials based electrodes for water desalination. J. Electroanal. Chem..

[cit2] Pearson J. L., Hegy M., Missimer T. M. (2021). Impacts of Feedwater Quality Change on the Oldest Continuously Operated Brackish-Water Reverse Osmosis Desalination Plant in the United States. Water.

[cit3] Fu C., Yi X., Gao Y. (2024). Effect of Electric Field on Membrane Fouling and Membrane Performance in Reverse Osmosis Treatment of Brackish Water. Appl. Sci..

[cit4] Philibert M., Villacorte L. O., Ekowati Y., Abushaban A., Salinas-Rodriguez S. G. (2024). Fouling and scaling in reverse osmosis desalination plants: A critical review of membrane autopsies, feedwater quality guidelines and assessment methods. Desalination.

[cit5] Ahualli S., Iglesias G. R., Delgado Á. V. (2018). Principles and Theoretical Models of CDI: Experimental Approaches. Interface Sci. Technol..

[cit6] Gamaethiralalage J. G. (2021). *et al.*, Recent advances in ion selectivity with capacitive deionization. Energy Environ. Sci..

[cit7] Vermaas D. A., Kunteng D., Saakes M., Nijmeijer K. (2013). Fouling in reverse electrodialysis under natural conditions. Water Res..

[cit8] Suss M. E., Porada S., Sun X., Biesheuvel P. M., Yoon J., Presser V. (2015). Water desalination *via* capacitive deionization: What is it and what can we expect from it?. Energy Environ. Sci..

[cit9] Welgemoed T. J., Schutte C. F. (2005). Capacitive Deionization Technology™: An alternative desalination solution. Desalination.

[cit10] Biesheuvel P. M., Van Limpt B., Van Der Wal A. (2009). Dynamic adsorption/desorption process model for capacitive deionization. J. Phys. Chem. C.

[cit11] Porada S., Zhao R., Van Der Wal A., Presser V., Biesheuvel P. M. (2013). Review on the science and technology of water desalination by capacitive deionization. Prog. Mater. Sci..

[cit12] AlMarzooqi F. A., Al Ghaferi A. A., Saadat I., Hilal N. (2014). Application of Capacitive Deionisation in water desalination: A review. Desalination.

[cit13] Cohen I., Avraham E., Soffer A., Aurbach D. (2013). Water desalination by capacitive deionization - advantages limitations and modification. ECS Trans..

[cit14] Gaikwad M. S., Balomajumder C. (2016). Capacitive deionization for desalination using nanostructured electrodes. Anal. Lett..

[cit15] Liu N. L., Chen L. I., Tsai S. W., Hou C. H. (2020). Enhanced desalination of electrospun activated carbon fibers with controlled pore structures in the electrosorption process. Environ. Sci..

[cit16] Liang P., Ren Z. J., Huang X. (2020). Capacitive deionization and electrosorption: from desalination to ion management. Environ. Sci.: Water Res. Technol..

[cit17] Chen R., Sheehan T., Ng J. L., Brucks M., Su X. (2020). Capacitive Deionization and Electrosorption for Heavy Metal Removal. Environ. Sci.: Water Res. Technol..

[cit18] Gabelich C. J., Tran T. D., Suffet I. H. (2002). Electrosorption
of inorganic salts from aqueous solution using carbon aerogels. Environ. Sci. Technol..

[cit19] Park K. H., Kwak D. H. (2014). Electrosorption and electrochemical properties of activated-carbon sheet electrode for capacitive deionization. J. Electroanal. Chem..

[cit20] Srimuk P. (2016). *et al.*, MXene as a novel intercalation-type pseudocapacitive cathode and anode for capacitive deionization. J. Mater. Chem. A.

[cit21] Elisadiki J., King’ondu C. K. (2020). Performance of ion intercalation materials in capacitive deionization/electrochemical deionization: A review. J. Electroanal. Chem..

[cit22] Singh K., Sahin S., Gamaethiralalage J. G., Zornitta R. L., de Smet L. C. P. M. (2022). Simultaneous, monovalent ion selectivity with polyelectrolyte multilayers and intercalation electrodes in capacitive deionization. Chem. Eng. J..

[cit23] Constantino V. R. L., Barbosa C. A. S., Bizeto M. A., Dias P. M. (2000). Intercalation Compounds involving Inorganic Layered Structures. An. Acad. Bras. Cienc..

[cit24] Cai Y., Wang Y., Zhang L., Fang R., Wang J. (2022). 3D Heterostructure Constructed by Few-Layered MXenes with a MoS2 Layer as the Shielding Shell for Excellent Hybrid Capacitive Deionization and Enhanced Structural Stability. ACS Appl. Mater. Interfaces.

[cit25] Stillwell A. S., Webber M. E. (2016). Predicting the specific energy consumption of reverse osmosis desalination. Water.

[cit26] Porada S. (2013). *et al.*, Direct prediction of the desalination performance of porous carbon electrodes for capacitive deionization. Energy Environ. Sci..

[cit27] Niu R., Li H., Ma Y., He L., Li J. (2015). An insight into the improved capacitive deionization performance of activated carbon treated by sulfuric acid. Electrochim. Acta.

[cit28] Gao X., Omosebi A., Landon J., Liu K. (2014). Enhancement of charge efficiency for a capacitive deionization cell using carbon xerogel with modified potential of zero charge. Electrochem. Commun..

[cit29] Liu P. (2017). *et al.*, Graphene-based materials for capacitive deionization, Royal Society of Chemistry. J. Mater. Chem. A.

[cit30] Li H., Gao Y., Pan L., Zhang Y., Chen Y., Sun Z. (2008). Electrosorptive desalination by carbon nanotubes and nanofibres electrodes and ion-exchange membranes. Water Res..

[cit31] Bao S., Duan J., Zhang Y. (2018). Characteristics of Nitric Acid-Modified Carbon Nanotubes and Desalination Performance in Capacitive Deionization. Chem. Eng. Technol..

[cit32] Tsouris C. (2011). *et al.*, Mesoporous carbon for capacitive deionization of saline water. Environ. Sci. Technol..

[cit33] Xu X. (2015). *et al.*, Flexible, highly graphitized carbon aerogels based on bacterial cellulose/lignin: Catalyst-free synthesis and its application in energy storage devices. Adv. Funct. Mater..

[cit34] Wen X., Zhang D., Shi L., Yan T., Wang H., Zhang J. (2012). Three-dimensional hierarchical porous carbon with a bimodal pore arrangement for capacitive deionization. J. Mater. Chem..

[cit35] Eliad L., Salitra G., Soffer A., Aurbach D. (2001). Ion sieving effects in the electrical double layer of porous carbon electrodes: Estimating effective ion size in electrolytic solutions. J. Phys. Chem. B.

[cit36] Porada S. (2013). *et al.*, Direct Prediction of the Desalination Performance
of Porous Carbons Electrodes for Capacitive Deionization. Energy Environ. Sci..

[cit37] Biesheuvel P. M., Hamelers H. V. M., Suss M. E. (2015). Theory of Water Desalination by Porous Electrodes with Immobile Chemical Charge. Colloids Interface Sci. Commun..

[cit38] Gao X., Porada S., Omosebi A., Liu K. L., Biesheuvel P. M., Landon J. (2016). Complementary surface charge for enhanced capacitive deionization. Water Res..

[cit39] Jo H., Kim K. H., Jung M. J., Park J. H., Lee Y. S. (2017). Fluorination effect of activated carbons on performance of asymmetric capacitive deionization. Appl. Surf. Sci..

[cit40] Andelman M. (2014). Ionic Group Derivitized Nano Porous Carbon Electrodes for Capacitive Deionization. J. Mater. Sci. Chem. Eng..

[cit41] Li L. X., Li F. (2011). The effect of carbonyl, carboxyl and hydroxyl groups on the capacitance of carbon nanotubes. N. Carbon Mater..

[cit42] Xu C. (2021). *et al.*, Prussian Blue Analogues in Aqueous Batteries and Desalination Batteries. Nano-Micro Lett..

[cit43] Chen J. (2020). *et al.*, Prussian blue, its analogues and their derived materials for electrochemical energy storage and conversion. Energy Storage Mater..

[cit44] Guo L. (2017). *et al.*, A Prussian blue anode for high performance electrochemical deionization promoted by the faradaic mechanism. Nanoscale.

[cit45] Zhang W. (2022). *et al.*, Well-dispersed Prussian blue analogues connected with carbon nanotubes for efficient capacitive deionization process. Sep. Purif. Technol..

[cit46] Torkamanzadeh M. (2020). *et al.*, MXene/Activated-Carbon Hybrid Capacitive Deionization for Permselective Ion Removal at Low and High Salinity. ACS Appl. Mater. Interfaces.

[cit47] Zhang Y., Zhang G., Zhao S., Gao A., Cui J., Yan Y. (2023). Three-Dimensional MXene-Based Functional Materials for Water Treatment: Preparation, Functional Tailoring, and Applications. Am. Chem. Soc..

[cit48] Zhang B., Boretti A., Castelletto S. (2022). Mxene Pseudocapacitive Electrode Material for Capacitive Deionization. Chem. Eng. J..

[cit49] Chen Y. W., Chen J. F., Lin C. H., Hou C. H. (2019). Integrating a supercapacitor with capacitive deionization for direct energy recovery from the desalination of brackish water. Appl. Energy.

[cit50] Gao X. (2023). *et al.*, Efficient hybrid capacitive deionization with MnO2/g-C3N4 heterostructure: Enhancing Mn dz2 electron occupancy by interfacial electron bridge for fast charge transfer. Desalination.

[cit51] Yao Q., Shi Z., Liu Q., Gu Z., Ning R. (2018). The influences of separators on capacitive deionization systems in the cycle of adsorption and desorption. Environ. Sci. Pollut. Res..

[cit52] Remillard E. M., Shocron A. N., Rahill J., Suss M. E., Vecitis C. D., Vecitis C. (2018). A Direct Comparison of Flow-By and Flow-Through Capacitive Deionization. Desalination.

[cit53] Linnartz C., Rommerskirchen A., Walker J., Plankermann-Hajduk J., Köller N., Wessling M. (2020). Membrane-electrode assemblies for flow-electrode capacitive deionization. J. Membr. Sci..

[cit54] Köller N. (2024). *et al.*, Comparison of current collector architectures for Flow-electrode Capacitive Deionization. Desalination.

[cit55] Ji H. (2014). *et al.*, Capacitance of carbon-based electrical double-layer capacitors. Nat. Commun..

[cit56] Nordstrand J. (2022). *et al.*, Ladder Mechanisms of Ion Transport in Prussian Blue Analogues. ACS Appl. Mater. Interfaces.

[cit57] Simonov A. (2020). *et al.*, Hidden diversity of vacancy networks in Prussian blue analogues. Nature.

[cit58] 34# COMPARING INTERCALATION ELECTRODES FOR ENERGY EFFICIEN Master_s_Thesis_-_Vineeth_Pothanamkandathil

[cit59] Murphy G. W., Caudle D. D. (1967). Mathematical theory of electrochemical demineralization in flowing systems. Electrochim. Acta.

[cit60] Johnson A. M., Newman J. (1971). Desalting by Means of Porous Carbon Electrodes. J. Electrochem. Soc..

[cit61] Flow through Patent Canadian

[cit62] Lee J. B., Park K. K., Eum H. M., Lee C. W. (2006). Desalination of a thermal power plant wastewater by membrane capacitive deionization. Desalination.

[cit63] Lee J., Kim S., Kim C., Yoon J. (2014). Hybrid capacitive deionization to enhance the desalination performance of capacitive techniques. Energy Environ. Sci..

[cit64] Il Jeon S. (2013). *et al.*, Desalination *via* a new membrane capacitive deionization process utilizing flow-electrodes. Energy Environ. Sci..

[cit65] Santos C. (2018). *et al.*, Interconnected metal oxide CNT fibre hybrid networks for current collector-free asymmetric capacitive deionization. J. Mater. Chem. A.

[cit66] Wunch M. A. (2024). *et al.*, Vanadium nitride-vanadium oxide-carbon nanofiber hybrids for high performance supercapacitors. Electrochim. Acta.

[cit67] Lado J. J. (2021). *et al.*, Performance analysis of a capacitive deionization stack for brackish water desalination. Desalination.

[cit68] Luo K. (2020). *et al.*, Desalination behavior and performance of flow-electrode capacitive deionization under various operational modes. Chem. Eng. J..

[cit69] Kang J., Kim T., Jo K., Yoon J. (2014). Comparison of salt adsorption capacity and energy consumption between constant current and constant voltage operation in capacitive deionization. Desalination.

[cit70] Kim T., Yoon J. (2015). CDI ragone plot as a functional tool to evaluate desalination performance in capacitive deionization. RSC Adv..

[cit71] Fan C. S., Liou S. Y. H., Hou C. H. (2017). Capacitive deionization of arsenic-contaminated groundwater in a single-pass mode. Chemosphere.

[cit72] Zhang F., Yang F. (2022). Single-pass capacitive deionization with a HNO3-modified electrode for fluoride removal. Environ. Eng. Res..

[cit73] Gendel Y., Rommerskirchen A. K. E., David O., Wessling M. (2014). Batch mode and continuous desalination of water using flowing carbon deionization (FCDI) technology. Electrochem. Commun..

[cit74] Jiang Y., Hou Z., Yan L., Gang H., Wang H., Chai L. (2022). A Novel Dual-Ion Capacitive Deionization System Design with Ultrahigh Desalination Performance. Polymers.

[cit75] Porada S., Weingarth D., Hamelers H. V. M., Bryjak M., Presser V., Biesheuvel P. M. (2014). Carbon flow electrodes for continuous operation of capacitive deionization and capacitive mixing energy generation. J. Mater. Chem. A.

[cit76] Hosseinzadeh M., Mozaffari S. A., Ebrahimi F. (2022). Porous 3D-graphene functionalized with MnO2 nanospheres and NiO nanoparticles as highly efficient electrodes for asymmetric capacitive deionization: Evaluation by impedance-derived capacitance spectroscopy. Electrochim. Acta.

[cit77] Gong A., Zhao Y., Liang B., Li K. (2022). Stepwise hollow Prussian blue/carbon nanotubes composite as a novel electrode material for high-performance desalination. J. Colloid Interface Sci..

[cit78] Beke M., Velempini T., Prabakaran E., Pillay K. (2023). Preparation of carbon-aerogel polypyrrole composite for desalination by hybrid capacitive desalination method. Arabian J. Chem..

[cit79] Huang W., Zhang Y., Bao S., Song S. (2013). Desalination by capacitive deionization with carbon-based materials as electrode: A review. Surf. Rev. Lett..

[cit80] Sharma V., Mishra S., raj S. K., Upadhyay P., Kulshrestha V. (2023). Carbon-based electrode materials with ion exchange membranes for enhanced membrane capacitive deionization. Colloids Surf., A.

[cit81] Su H., Lin S., Deng S., Lian C., Shang Y., Liu H. (2019). Predicting the capacitance of carbon-based electric double layer capacitors by machine learning. Nanoscale Adv..

[cit82] Noked M., Soffer A., Arubach D. (2011). The electrochemistry of activated carbonaceous materials: Past, present, and future. J. Solid State Electrochem..

[cit83] Mehek R., Iqbal N., Noor T., Wang Y., Ganin A. Y. (2023). Efficient electrochemical performance of MnO2 nanowires interknitted vanadium oxide intercalated nanoporous carbon network as cathode for aqueous zinc ion battery. J. Ind. Eng. Chem..

[cit84] Kajiyama S. (2016). *et al.*, Sodium-Ion Intercalation Mechanism in MXene Nanosheets. ACS Nano.

[cit85] Liu R., Yao S., Shen Y. (2022). Pore-scale study of ion transport and intercalation processes of capacitive deionization cells with intercalation electrodes based on lattice Boltzmann method. Desalination.

[cit86] Zhang Y. (2022). *et al.*, MOF-on-MOF Nanoarchitectonics for Selectively Functionalized Nitrogen-Doped Carbon-Graphitic Carbon/Carbon Nanotubes 2 Heterostructure with High Capacitive Deionization Performance 3 4. Nano Energy.

[cit87] Zhuang R. (2020). *et al.*, Acidified bamboo-derived activated carbon/manganese dioxide composite as a high-performance electrode material for capacitive deionization. Int. J. Electrochem. Sci..

[cit88] Yin Q. (2023). *et al.*, MnO2-modified soybean root derived porous carbon with excellent capacity deionization. Diamond Relat. Mater..

[cit89] Xu L. (2023). *et al.*, Carbon nanotube bridged nickel hexacyanoferrate architecture for high-performance hybrid capacitive deionization. J. Colloid Interface Sci..

[cit90] Lei Y., Wang S., Zhao L., Li C., Wang G., Qiu J. (2024). Entropy Engineering Constrain Phase Transitions Enable Ultralong-life Prussian Blue Analogs Cathodes. Adv. Sci..

[cit91] Wang S. (2020). *et al.*, In Situ Formation of Prussian Blue Analogue Nanoparticles Decorated with Three-Dimensional Carbon Nanosheet Networks for Superior Hybrid Capacitive Deionization Performance. ACS Appl. Mater. Interfaces.

[cit92] Li J. (2024). *et al.*, Enhanced redox kinetics of Prussian blue analogues for superior electrochemical deionization performance. Chem. Sci..

[cit93] Wang S. (2024). *et al.*, Enabling High Capacity and Stable Sodium Capture in Simulated Saltwater by Highly Crystalline Prussian Blue Analogues Cathode. Small Struct..

[cit94] Ma D. (2024). *et al.*, Improved Na+ adsorption performance and storage mechanism of cobalt hexacyanoferrate/polyaniline composite during the hybrid capacitive deionization process. Sep.
Purif. Technol..

[cit95] Wang X. (2024). *et al.*, Prussian Blue/Carbon Nanofiber Amalgamated Conductive Scaffolds for Capacitive Deionization. ACS Appl. Nano Mater..

[cit96] Santos C. (2018). Interconnected metal oxide CNT fibre hybrid networks for current collector-free asymmetric capacitive deionization. J. Mater. Chem. A.

[cit97] Yin H. (2013). *et al.*, Three-dimensional graphene/metal oxide nanoparticle hybrids for high-performance capacitive deionization of saline water. Adv. Mater..

[cit98] Tan G., Wan S., Mei S. C., Gong B., Qian C., Chen J. J. (2023). Boosted brackish water desalination and water softening by facilely designed MnO2/hierarchical porous carbon as capacitive deionization electrode. Water Res. X.

[cit99] Liu B., Yu L., Yu F., Ma J. (2021). In-situ formation of uniform V2O5 nanocuboid from V2C MXene as electrodes for capacitive deionization with higher structural stability and ion diffusion ability. Desalination.

[cit100] Srimuk P. (2016). *et al.*, MXene as a novel intercalation-type pseudocapacitive cathode and anode for capacitive deionization. J. Mater. Chem. A.

[cit101] Li B. (2022). *et al.*, Tailoring interlayer spacing in MXene cathodes to boost the desalination performance of hybrid capacitive deionization systems. Nano Res..

[cit102] Chen Z., Xu X., Wang K., Meng F., Lu T., Pan L. (2023). Dual-confinement effect in metal oxide nanoparticles/MXene-reduced graphene oxide for high capacitive deionization performance. Desalination.

[cit103] Tan Z. (2023). *et al.*, Ti3C2Tx MXene@carbon dots hybrid microflowers as a binder-free electrode material toward high capacity capacitive deionization. Desalination.

[cit104] Han J., Yan T., Shen J., Shi L., Zhang J., Zhang D. (2019). Capacitive Deionization of Saline Water by Using MoS2-Graphene Hybrid Electrodes with High Volumetric Adsorption Capacity. Environ. Sci. Technol..

[cit105] Cai Y., Zhang W., Fang R., Zhao D., Wang Y., Wang J. (2021). Well-dispersed few-layered MoS2 connected with robust 3D conductive architecture for rapid capacitive deionization process and its specific ion selectivity. Desalination.

[cit106] Li L., Xu S., Carter E. A. (2020). First-Principles Modeling of Sodium Ion and Water Intercalation into Titanium Disulfide Interlayers for Water Desalination. Chem. Mater..

[cit107] Wang Z. (2019). *et al.*, Nanoarchitectured metal-organic framework/polypyrrole hybrids for brackish water desalination using capacitive deionization. Mater. Horiz..

[cit108] Xu X. (2020). *et al.*, Ultrahigh capacitive deionization performance by 3D interconnected MOF-derived nitrogen-doped carbon tubes. Chem. Eng. J..

[cit109] Zhang J., Fang J., Han J., Yan T., Shi L., Zhang D. (2018). N, P, S co-doped hollow carbon polyhedra derived from MOF-based core-shell nanocomposites for capacitive deionization. J. Mater. Chem. A.

[cit110] Wang N., Zhang Y., Hu T., Zhao Y., Meng C. (2015). Facile hydrothermal synthesis of ultrahigh-aspect-ratio V2O5 nanowires for high-performance supercapacitors. Curr. Appl. Phys..

[cit111] Zhang Z., Wang J. G., Wei B. (2017). Facile synthesis of mesoporouscobalt hexacyanoferrate nanocubes for high-performance supercapacitors. Nanomaterials.

[cit112] Yuan B., Sun F., Li C., Huang W., Lin Y. (2019). Formation of Prussian blue analog on Ni foam *via in situ* electrodeposition method and conversion into Ni-Fe-mixed phosphates as efficient oxygen evolution electrode. Electrochim. Acta.

[cit113] Kim C., Lee J., Kim S., Yoon J. (2014). TiO2 sol-gel spray method for carbon electrode fabrication to enhance desalination efficiency of capacitive deionization. Desalination.

[cit114] Influence of particle size distribution on carbon

[cit115] Jin J. (2020). *et al.*, Phase- And Crystallinity-Tailorable MnO2as an Electrode for Highly Efficient Hybrid Capacitive Deionization (HCDI). ACS Sustain. Chem. Eng..

[cit116] Yang K. (2024). *et al.*, Synthesis by design and application to desalination of VO2/CTAB-Ti3C2 capacitive deionization electrode. J. Electroanal. Chem..

[cit117] Li Y., Liu H., Peng W., Li Y., Zhang F., Fan X. (2023). Plasma-enhanced vanadium-based hybrid capacitive deionization for high selective removal of Pb2+. Desalination.

[cit118] Vafakhah S., Saeedikhani M., Ding M., Guo L., Von Lim Y., Yang H. Y. (2022). Size and composition regulated sodium vanadium fluorophosphate wrapped in rGO as an efficient cathode for brackish and seawater desalination. Desalination.

[cit119] Porada S., Shrivastava A., Bukowska P., Biesheuvel P. M., Smith K. C. (2017). Nickel Hexacyanoferrate Electrodes for Continuous Cation Intercalation Desalination of Brackish Water. Electrochim. Acta.

[cit120] Lee J., Kim S., Yoon J. (2017). Rocking Chair Desalination Battery Based on Prussian Blue Electrodes. ACS Omega.

[cit121] Gong A., Zhao Y., He M., Liang B., Li K. (2021). High-performance desalination of three-dimensional nitrogen-doped carbon framework reinforced Prussian blue in capacitive deionization. Desalination.

[cit122] Sabzi R. E., Kant K., Losic D. (2010). Electrochemical synthesis of nickel hexacyanoferrate nanoarrays with dots, rods and nanotubes morphology using a porous alumina template. Electrochim. Acta.

[cit123] Martinez Joaristi A., Juan-Alcañiz J., Serra-Crespo P., Kapteijn F., Gascon J. (2012). Electrochemical synthesis of some archetypical Zn 2+, Cu 2+, and Al 3+ metal organic frameworks. Cryst. Growth Des..

[cit124] Phuoc N. M. (2020). *et al.*, Enhanced desalination performance of capacitive deionization using nanoporous carbon derived from zif-67 metal organic frameworks and cnts. Nanomaterials.

[cit125] Arulrajan A. C., Dykstra J. E., Van Der Wal A., Porada S. (2021). Unravelling pH Changes in Electrochemical Desalination with Capacitive Deionization. Environ. Sci. Technol..

[cit126] Lumley M. A., Nam D. H., Choi K. S. (2020). Elucidating structure–composition–property relationships of Ni-based Prussian blue analogues for electrochemical seawater desalination. ACS Appl. Mater. Interfaces.

[cit127] Ahn J., Joo H., Il Jeon S., Yoon J., Lee J. (2024). High capacity rocking-chair capacitive deionization using highly crystalline sodium cobalt hexacyanoferrate (NaCoHCF) electrodes. Environ. Eng. Res..

[cit128] Taha M. M. (2023). *et al.*, Exceptional long-term stability of titanium oxynitride nanoparticles as non-carbon-based electrodes for aerated saline water capacitive deionization. Desalination.

[cit129] Chamoun M., Brant W. R., Tai C. W., Karlsson G., Noréus D. (2018). Rechargeability of aqueous sulfate Zn/MnO2 batteries enhanced by accessible Mn2+ ions. Energy Storage Mater..

[cit130] Liu M., Xue Z., Zhang H., Li Y. (2021). Dual-channel membrane capacitive deionization based on asymmetric ion adsorption for continuous water desalination. Electrochem. Commun..

[cit131] Liu J. (2022). *et al.*, MnO_2_-based Materials for Supercapacitor Electrodes: Challenges, Strategies and Prospects. RSC Adv..

[cit132] Ma X., Chen Y. A., Zhou K., Wu P. C., Hou C. H. (2019). Enhanced desalination performance *via* mixed capacitive-Faradaic ion storage using RuO2-activated carbon composite electrodes. Electrochim. Acta.

[cit133] Stock N., Biswas S. (2012). Synthesis of metal-organic frameworks (MOFs): Routes to various MOF topologies, morphologies, and composites. Chem. Rev..

[cit134] Vaitsis C. , Sourkouni G., and Argirusis C., Sonochemical synthesis of MOFs, in Metal-Organic Frameworks for Biomedical Applications, Elsevier, 2020, pp. 223–244. doi: 10.1016/B978-0-12-816984-1.00013-5

[cit135] Cai D. (2015). *et al.*, Rational synthesis of metal-organic framework composites, hollow structures and their derived porous mixed metal oxide hollow structures. J. Mater. Chem. A.

[cit136] Choi W. S., Lee H. J. (2022). Nanostructured Materials for Water Purification: Adsorption of Heavy Metal Ions and Organic Dyes. Polymers.

[cit137] Wang K., Liu Y., Ding Z., Li Y., Lu T., Pan L. (2019). Metal-organic-frameworks-derived NaTi2(PO4)3/carbon composites for efficient hybrid capacitive deionization. J. Mater. Chem. A.

[cit138] Shi W. (2021). *et al.*, Achieving Enhanced Capacitive Deionization by Interfacial Coupling in PEDOT Reinforced Cobalt Hexacyanoferrate Nanoflake Arrays. Glob. Chall..

[cit139] Shen X., Xiong Y., Yu F., Ma J. (2023). Chinese dumpling-like NaTi2(PO4)3/MXene@reduced graphene oxide for capacitive deionization with high capacity and good cycling stability. J. Mater. Chem. A.

[cit140] Yu L., Liu N., Liu B., Yu F., Ma J. (2023). In-situ-derived carbon coated sea urchin-like Na_3_V_2_(PO_4_)_3_ from V_2_C MXene for high-performance capacitive deionization. J. Alloys Compd..

[cit141] Cao Y., Yan L., Gang H., Wu B., Wei D., Wang H. (2023). Large gap cobalt-vanadium oxide structure encapsulated in porous carbon for high performance capacitive deionization. Sep. Purif. Technol..

[cit142] Liu S. (2023). *et al.*, Uniformly Dispersed Fe-N Active Centers on Hierarchical Carbon Electrode for High-Performance Capacitive Deionization: Plentiful Adsorption Sites and Conductive Electron Transfer. ACS Sustain. Chem. Eng..

[cit143] Ahmed F. (2023). *et al.*, Manganese dioxide nanoparticles/reduced graphene oxide nanocomposites for hybrid capacitive desalination. Adv. Compos. Hybrid Mater..

[cit144] Baburaj A. (2020). *et al.*, Multilayer graphene coated vanadium(V) oxide as electrodes for intercalation based brackish water desalination. 2D Mater..

[cit145] Zhao J., Zhao Z., Sun Y., Ma X., Ye M., Wen X. (2021). 3D hierarchical porous N-doped carbon quantum dots/vanadium nitride hybrid microflowers as a superior electrode material toward high-performance asymmetric capacitive deionization. Environ. Sci.:Nano.

